# Transsynaptic Binding of Orphan Receptor GPR179 to Dystroglycan-Pikachurin Complex Is Essential for the Synaptic Organization of Photoreceptors

**DOI:** 10.1016/j.celrep.2018.08.068

**Published:** 2018-10-02

**Authors:** Cesare Orlandi, Yoshihiro Omori, Yuchen Wang, Yan Cao, Akiko Ueno, Michel J. Roux, Giuseppe Condomitti, Joris de Wit, Motoi Kanagawa, Takahisa Furukawa, Kirill A. Martemyanov

**Affiliations:** 1 Department of Neuroscience, The Scripps Research Institute, Jupiter, FL 33458, USA; 2 Laboratory for Molecular and Developmental Biology, Institute for Protein Research, Osaka University, Osaka, Japan; 3 Institut de Ge´ne´ tique et de Biologie Mole´ culaire et Cellulaire, Universite´ de Strasbourg, Centre National de la Recherche Scientifique, UMR7104, INSERM, U1258, Illkirch, France; 4 VIB Center for Brain & Disease Research, Herestraat 49, 3000 Leuven, Belgium; 5 KU Leuven, Department of Neurosciences, Herestraat 49, 3000 Leuven, Belgium; 6 Division of Molecular Brain Science, Kobe University Graduate School of Medicine, Kobe 650-0017, Japan; 7 Lead Contact

## Abstract

Establishing synaptic contacts between neurons is paramount for nervous system function. This process involves transsynaptic interactions between a host of cell adhesion molecules that act in cooperation with the proteins of the extracellular matrix to specify uniquephysiological propertiesofindividual synaptic connections. However, understanding of the molecular mechanisms that generate functional diversity in an input-specific fashion is limited. In this study, we identify that major components of the extracellular matrix proteins present in the synaptic cleft—members oftheheparansulfateproteoglycan (HSPG) family—associate with the GPR158/179 group of orphan receptors. Using the mammalian retina as a model system, we demonstrate that the HSPG member Pikachurin, released by photoreceptors, recruits a key post-synaptic signaling complex of downstream ON-bipolar neurons in coordination with the presynaptic dystroglycan glycoprotein complex. We further demonstrate that this transsynaptic assembly plays an essential role in synaptic transmission of photoreceptor signals.

## In Brief

Orlandi et al. identify transsynaptic assembly at photoreceptor synapses involving pre-synaptic dystrophindystroglycan complex and the postsynaptic orphan receptor GPR179 bridged by HSPG protein Pikachurin in the cleft and demonstrate its role in shaping transmission of photoreceptor signals.

## Graphical Abstract

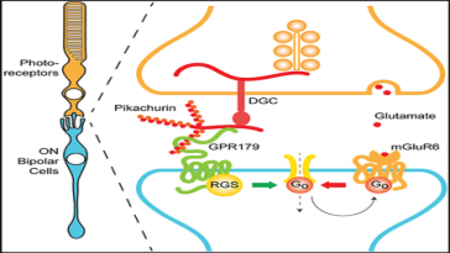

## INTRODUCTION

Precise synaptic connectivity is one of the defining properties of the CNS. The ability of neurons to form synapses with an extremely defined spatial and temporal resolution is essential to establish functional neuronal circuits, but the molecular mechanisms involved in neuronal wiring specificity are still poorly understood. To establish proper connections, a network of transsynaptic interactions among membrane receptors, secreted ligands, and synaptic cell adhesion molecules coordinates preand post-synaptic assembly (Chia et al., 2013; Sanes and Yamagata, 2009; Siddiqui and Craig, 2011). Beyond a structural role, several components of the extracellular matrix (ECM) have been shown to play an active role in the formation and maintenance of correct synaptic connectivity (de Wit et al., 2013; Dityatev et al., 2010; Nitkin et al., 1987).

Members of the G protein-coupled receptor (GPCR) family are among the most common resident proteins present at synapses. A wide variety of extracellular domains allows this large receptor family to sense a range of changes in the extracellular environment, including detection of all known neurotransmitters (Rosenbaum et al., 2009). Traditionally, GPCRs have been considered powerful modulators of neurotransmission that shape properties of neuronal circuits (Bargmann and Marder, 2013; Marder, 2012). However, emerging proteomic studies increasingly point to their involvement in transsynaptic macromolecular complexes and interactions with ECM components (Bolliger et al., 2011; Cao et al., 2015; Kakegawa et al., 2015; Lanoue et al., 2013; Luo et al., 2011; O’Sullivan et al., 2012). Such effects were primarily shown for the subfamily of “adhesion” receptors, and the scope of this involvement and extent of conservation across the GPCR superfamily are yet to be explored.

Functional roles and signal transduction mechanisms of a large portion of the GPCR family remain poorly understood, with many receptors still “orphan” of endogenous ligands. Nonetheless, genomic studies in humans and the use of knockout animal models suggest a crucial role for the largely unexplored biology of orphan receptors in fundamental neuronal processes (Ahmad et al., 2015; Kroeze et al., 2015). Our progress in de-orphanizing these receptors and understanding their physiology has been slow, likely because of their unusual biology, which may deviate from the traditional role of GPCRs as mediators of neurotransmitter signaling.

One of the classical models for studying synaptic organization whereby traditional and orphan GPCRs cooperate is offered by the first visual synapse of vertebrate photoreceptors. In the dark, photoreceptors tonically release the neurotransmitter glutamate, which is sensed by the mGluR6 receptor on the post-synaptic neuron: the ON-bipolar cell (ON-BC). The mGluR6 initiates a prototypic GPCR cascade that activates the G protein Gao to keep the effector channel TRPM1 inhibited (Koike et al., 2010; Morgans et al., 2009; Shen et al., 2009). Suppression of the glutamate release by light leads to TRPM1 opening and requires rapid inactivation of Gao. This is achieved by the action of the GTPase activating protein (GAP) complex, which involves coordinated action of several proteins, including catalytic subunits RGS7 and RGS11 (Martemyanov and Sampath, 2017; Vardi and Dhingra, 2014). The abundance and subcellular localization of the GAP complex have a major impact on the synaptic transmission of light signal from photoreceptors to ON-BC and tuning the circuits for daylight and dim vision (Cao et al., 2009; Sarria et al., 2015). A critical role in this process belongs to the orphan receptor GPR179, which has been identified as a component of the GAP complex serving in a non-traditional capacity as membrane anchor for RGS proteins at the ON-BC post-synaptic site (Orlandi et al., 2012). Knockout of GPR179 prevents postsynaptic accumulation of RGS proteins and severely compromises synaptic communication with photoreceptors (Orlandi et al., 2012; Peachey et al., 2012), indicating that it is required for achieving temporal resolution needed for a rapid transduction of visual stimuli. However, the mechanisms of synaptic targeting of GPR179 and its integration into the synaptic architecture remain unknown. Although GPR179 is largely retina specific, its close homolog GPR158 is enriched in the brain, where it likewise plays a role in organizing RGS complexes (Orlandi et al., 2012; Orlandi et al., 2015). Both proteins feature large extracellular segments, suggesting that they may be involved in the interactions with the ECM (Orlandi et al., 2012; Patel et al., 2013). In fact, ECM plays an essential role in the organization of the first visual synapse. One of the most prominent examples is provided by the complex of a/b-dystroglycan-dystrophins (DGCs) with the ECM protein Pikachurin, which is required for proper development and neurotransmission at the synapse (Omori et al., 2012; Sato et al., 2008). Notably, ablation of Pikachurin in mice results in ultrastructural abnormalities of the photoreceptor synapse and deficits in synaptic transmission. However, it is unclear how the photoreceptor Pikachurin-DGC complex engages ON-BCs and what its post-synaptic molecular targets are.

Here we identify ECM components heparan sulfate proteoglycans (HSPGs) as interaction partners of the orphan GPCRs, GPR179 and GPR158, and demonstrate an essential role of these interactions in synaptic targeting. Using the first visual synapse as a model, we provide evidence that the photoreceptor-released HSPG Pikachurin dictates the post-synaptic organization of the GAP complex by anchoring GPR179-RGS at the dendritic tips of ON-BCs. We further show that this function involves transsynaptic interaction with the DGC at the axonal terminals and that its disruption alters synaptic neurotransmission of photoreceptors in a process that involves RGS protein recruitment.

## RESULTS

### HSPGs Are Extracellular Binding Partners of GPR158 and GPR179 Receptors

**Figure 1.**
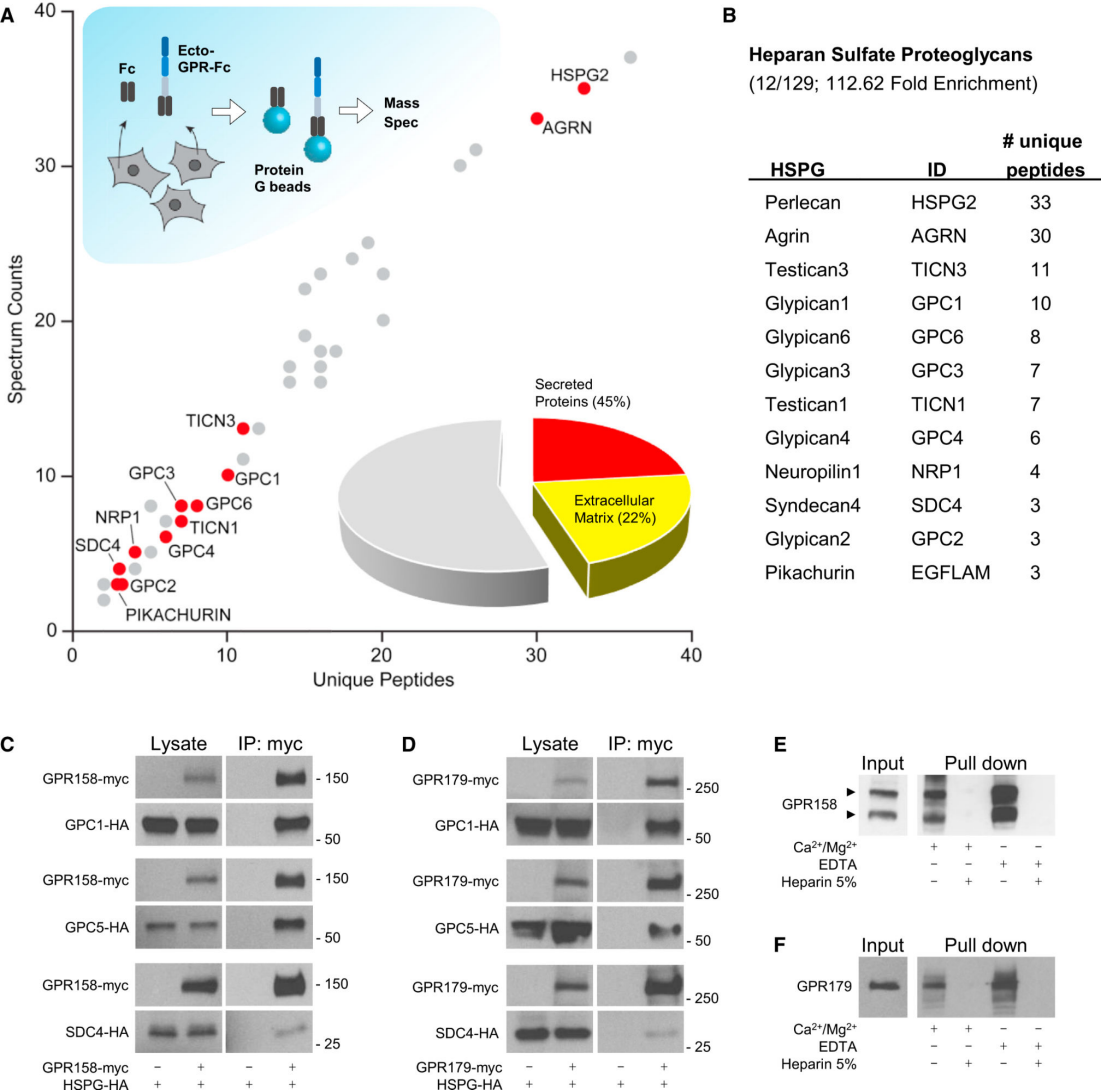
Unbiased Identification of GPR158 and GPR179 as Membrane Receptors for Heparan Sulfate Proteoglycans. (A and B) HEK293 cells were transfected with ecto-GPR158-Fc or Fc as negative control (A). The Fc proteins released in the media were isolated by incubation with protein G beads and analyzed using liquid chromatography tandem mass spectrometry (LC-MS/MS). The graph represents the interacting proteins identified specifically in the ecto-GPR-Fc pull-down experiment. DAVID analysis of the full set of identified proteins revealed the presence of 45% of secreted proteins, half of which are ECM components. The family of HSPGs is highlighted because enriched by the pull-down, and the identified members are listed in the table (B). (C and D) *In vitro* co-immunoprecipitation of GPR158 (C) and GPR179 (D) with representative HSPGs: GPC1, GPC5, and SDC4. HEK293 cells were transfected with the indicated myc- or HA-tagged constructs. Immunoprecipitated proteins were detected by western blotting using specific antibodies. Cells transfected with only HSPGs served as a control for non-specific binding. (E) Heparin-Sepharose pull-down from brain extract and western blot detection using a GPR158-specific antibody. Divalent cations or EDTA did not affect thepull-down. An excess of heparin (5%) was used as negative control. (F) Heparin-Sepharose pull-down from retina extract and western blot of GPR179. Divalent cations or EDTA did not affect the pull-down. An excess of heparin(5%) was used as negative control.

Orphan receptors GPR158 and GPR179 contain large extracellular segments that feature an EGF-like Ca^2+^-binding domain and a leucine repeat sequence (Orlandi et al., 2012; Patel et al., 2013), suggesting their possible role in association with ECM proteins. To test this possibility, we conducted an unbiased proteomics search for their extracellular binding partners (Figure 1A) in HEK293 cells known to express a wide range of receptors, cell adhesion molecules, and matrix proteins (Geiger et al., 2012; Lin et al., 2014; Thomas and Smart, 2005). Given high sequence homology between GPR158 and GPR179 (Figure S1A), our initial experiments were conducted with the N-terminal ectodomain of GPR158 fused to a human IgG Fc fragment (ecto-GPR158-Fc) directing its secretion to the medium. Following transfection of HEK293 cells with the ectoGPR158-Fc, secreted proteins were purified using protein G beads that captured Fc fragments. In parallel, the same experiment was conducted with Fc construct alone and used as a negative control to assess non-specific binding. Mass spectrometric identification of the proteins eluted from the beads identified 129 proteins specifically co-isolated with the ectoGPR158-Fc but not with the Fc fragment (Figure 1A). A Gene Ontology analysis of the data revealed that about half of these proteins were classified as secreted, with the largest group (21.71%) constituting ECM components. Remarkably, the most abundant proteins in this group belonged to a family of proteoglycans post-translationally modified by HS, classified as HSPGs (Bishop et al., 2007). In total, we found 12 different HSPGs specifically co-purified with GPR158 ectodomain (Figures 1B and S1B).

To confirm the interactions, we studied binding of several representative HSPG members to full-length GPR158 by coimmunoprecipitation upon co-expression in HEK293 cells (Figure 1C). We found a robust pull-down of all tested HSPGs by GPR158. This interaction was specific, as no binding was detected upon omitting the bait protein from the transfection. Reciprocal experiments similarly revealed effective and specific pull-down of GPR158 when HSPGs were used as baits (Figure S1C).

We next tested whether GPR179 could also bind to HSPGs given considerable sequence conservation between the extracellular domains of GPR158 and GPR179 (Figure S1A). These experiments were designed similarly, and the interaction was first studied upon co-transfection of epitope-tagged full-length GPR179 and candidate HSPGs into HEK293 cells. Again, we found that immunoprecipitation of GPR179 specifically pulled down all of the HSPGs tested (Figure 1D). The binding was further confirmed in the reverse direction where HSPGs were also able to pull down GPR179 (Figure S1D).

The ubiquitous nature of GPR158/179 interactions with various HSPG members, which do not share extensive homology in their amino acid sequences, prompted us to evaluate the role of HS chains in binding. In these experiments, we used beads directly conjugated to heparin, a highly sulfated form of HS often used in affinity chromatography to isolate HS-binding proteins (Ori et al., 2011). Indeed, we found that heparin beads were able to effectively pull down native GPR158 from a membrane-enriched brain lysate. The interaction was specific, as inclusion of excess free unbound heparin prevented GPR158 retention by the beads. We further examined the role of divalent cations in the interaction, given their role in regulating many extracellular interactions (Maurer and Hohenester, 1997) and the presence of the putative Ca^2+^-binding motif in GPR158/ 179 (Orlandi et al., 2012; Patel et al., 2013). We found that retention of GPR158 by heparin beads was insensitive to either addition of excess Ca^2+^/Mg^2+^ or their chelation by EDTA (Figure 1E). Native GPR179 exhibited similar behavior, suggesting that divalent cations may not be involved in modulating this interaction (Figure 1F). Together, these results establish HSPGs as extracellular binding partners of GPR158/179 orphan receptors and reveal sufficiency of HS side chains for the interaction.

### Pikachurin Is the Endogenous HSPG Client of GPR179 in Photoreceptor Synapses

**Figure 2.**
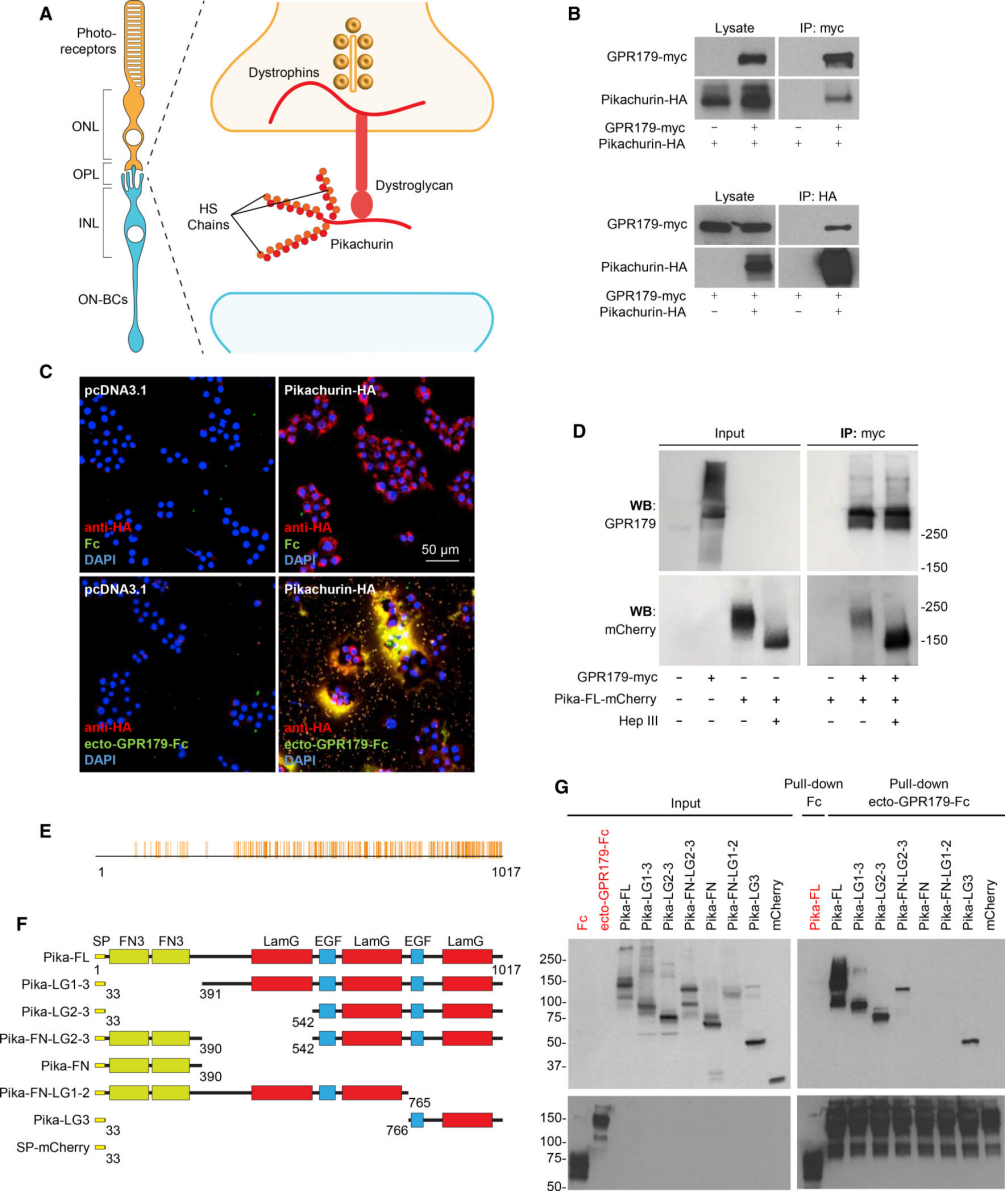
Characterization of Interactions between GPR179 and the Photoreceptor-Released HSPG Pikachurin In Vitro. (A) Scheme of the pre-synaptic compartment at the first visual synapse. (B) *In vitro* co-immunoprecipitation of GPR179 with Pikachurin in HEK293 cells transfected with the indicated myc- or HA-tagged constructs. Immunoprecipitated proteins were detected by western blotting using specific antibodies. (C) Live staining of Pikachurin (red) in transfected HEK293 cells shows a predominant localization within the extracellular matrix. Incubation with conditionedmedia of HEK293 cells expressing the ecto-GPR179-Fc (green) shows co-localization with the anti-HA antibody staining (red). Negative controls are cells transfected with empty vector (pcDNA3.1) or incubated with Fc fragment. DAPI in blue. (D) Heparan sulfate modification of Pikachurin is not required for the interaction with GPR179. GPR179-myc was immunoprecipitated from cell lysate oftransfected HEK293 cells. GPR179-conjugated beads were mixed with conditioned media from cells transfected with Pikachurin-mCherry and treated with heparinase III or buffer. Cells transfected with empty vector were used as immunoprecipitation (IP) specificity control. (E) Multiple amino acid sequence alignment of pikachurin from 197 species. Identical amino acids are highlighted in orange and aligned with domain topology(below). (F) Schematics of Pikachurin structural domains and deletion constructs generated to study binding determinants to GPR179. Each construct is fused with mCherry at its C terminus. Amino acid numbers are shown at the bottom of each construct. (G) Pull-down of Pikachurin-derived mutants from HEK293 cell lysates by GPR179 ectodomain fused to an Fc fragment. Fc and mCherry are used as negativecontrols.

To explore physiological relevance of the GPR158/179-HSPG interactions in the context of native neuronal circuits, we turned our attention to one particular candidate HSPG identified in the screen: an Agrin-like protein, Pikachurin, one of the least characterized members of the family (Manabe et al., 2008). Pikachurin is specifically expressed in the retina by both rod and cone photoreceptors and released in the synaptic cleft, where it has been identified as a ligand for the pre-synaptic DGC (Sato et al., 2008) (Figure 2A). Intriguingly, the function of photoreceptor synapses requires the presence of GPR179, expressed by the postsynaptic ON-BC and targeted to the dendritic tips in apposition to pre-synaptic release sites of both rods and cones (Audo et al., 2012; Peachey et al., 2012). In contrast, brain-enriched GPR158 is not detected in either photoreceptors or ON-BC (Sarin et al., 2018; Shekhar et al., 2016), making GPR179 at this synapse non-redundant. First, we confirmed the interaction between GPR179 and Pikachurin in transfected HEK293 cells, where we detected robust and specific co-immunoprecipitation of both proteins in both forward and reverse directions (Figure 2B). We further established that the binding is mediated by the ectodomain of GPR179 using an overlay approach. A live staining of Pikachurin upon transfection in HEK293 cells revealed its predominant extracellular localization within the ECM (Figure 2C). These Pikachurin-positive patches were stained by application of ecto-GPR179-Fc (Figure 2C). The interaction of the ectoGPR179 and Pikachurin in the ECM was specific, as no staining was observed when Fc carrier alone was used or when Pikachurin was omitted from the transfection, indicating low expression of endogenous Pikachurin in HEK293 cells, which was nevertheless detectable by mass spectrometry in our proteomic screen. Because HS side chains were sufficient for interaction with GPR158/179 (Figure 1E), we next asked a converse question: whether HS modification of Pikachurin is the sole requirement for GPR179 binding. Enzymatic treatment with heparinase III resulted in a clear mobility shift of Pikachurin isolated from HEK293 medium, confirming its modification with HS side chains (Figure 2D). However, Pikachurin stripped from HS still effectively co-immunoprecipitated with GPR179, indicating the involvement of additional binding sites on Pikachurin for the interaction with GPR179 (Figure 2D). Like other secreted members of the HSPG family, Pikachurin contains several conserved structural domains (Sato et al., 2008) that can possibly be involved in HS-independent binding to GPR179. Multiple sequence alignment across 197 species revealed a high degree of amino acid conservation across the three laminin G and the two EGF-like domains in the C terminus of the protein, with the highest similarity (55.5%) in the third laminin G domain (Figure 2E). Accordingly, we generated several deletion constructs lacking various conserved domains of Pikachurin (Figure 2F). In these experiments, we used cell lysates in which HS modification does not occur to specifically analyze contribution of protein moiety to binding. Pull-down experiments with ecto-GPR179-Fc as a bait and the Fc carrier as negative control revealed that the C-terminal 251 amino acids (Pika-LG3) were both necessary and sufficient for the interaction with GPR179 (Figure 2G). Thus, we conclude that Pikachurin, when secreted in the ECM, specifically interacts with the ectodomain of GPR179 receptor via multiple sites including the HS side chains and the LG3 domain of the protein core.

**Figure 3.**
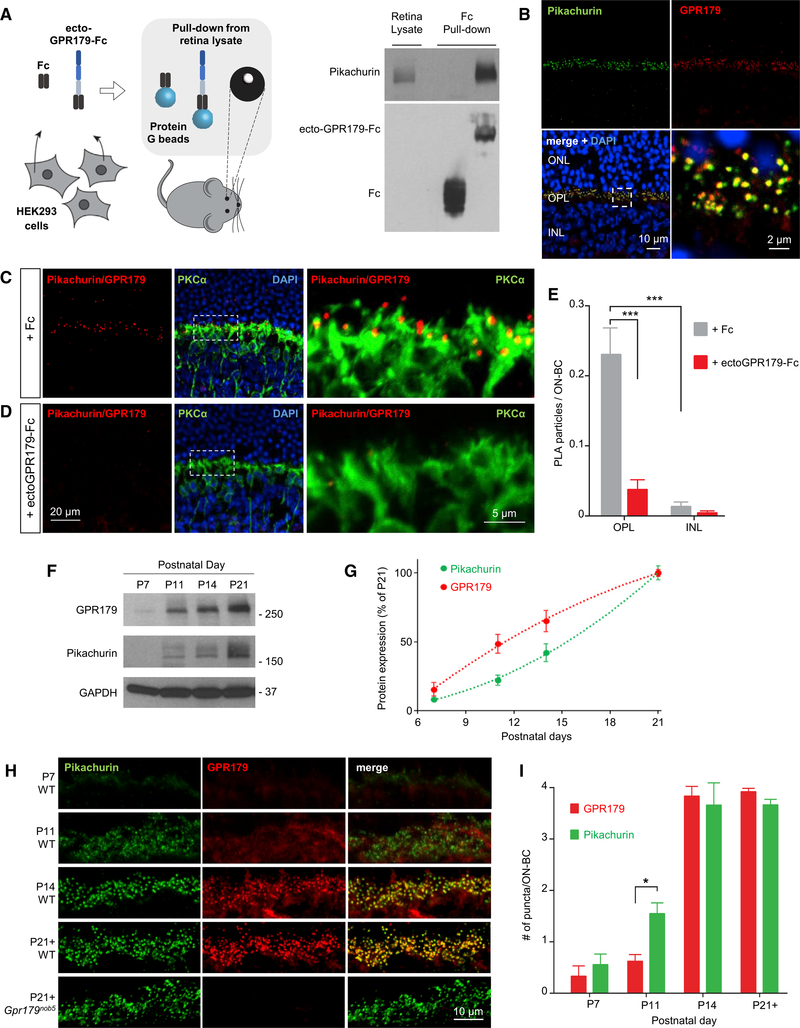
Analysis of GPR179-Pikachurin Complex Formation in the Retina. (A) Scheme of the experimental paradigm (left). Pull-down assay of endogenous Pikachurin from retina lysates using affinity-purified ecto-GPR179-Fc expressedin transfected HEK293 cells. Fc fragment was used as negative control. (B) Confocal images of retina sections show co-localization of Pikachurin (green) and GPR179 (red) in the retina OPL. DAPI in blue. (C and D) Detection of the complex GPR179/Pikachurin (red) using proximity ligation assay (PLA) in retina cross-sections from wild-type mice. Primary antibodies were co-incubated with conditioned media of HEK293 cells expressing either the Fc fragment (C) or the ecto-GPR179-Fc as negative control (D). Dashed-line boxes indicate the region of the merged image reported with a higher magnification. PKCα (green) was used as a marker of ON-BC, and nuclei were labeled by DAPI (blue). (E) Quantification of PLA particles in the OPL or inner nuclear layer (INL) (negative control) of retina cross-sections in each condition. Data are mean ± SEM (n = 5–7, ***p < 0.001, Student’s t test). (F and G) Representative western blots (F) and quantification (G) of Pikachurin and GPR179 protein levels in retina lysates at different developmental stages. Equal amounts of total protein were loaded on a single gel, and specific antibodies were used to detect indicated proteins (n = 3). (H) Immunohistochemistry (IHC) of WT retinas showing expression pattern of Pikachurin (green) and GPR179 (red) at different developmental stages. Immunostaining of adult *Gpr179*^*nob5*^ retina (bottom) shows similar Pikachurin accumulation at synaptic sites. (I) Quantification of GPR179- and Pikachurin-positive puncta at the OPL during retina development in WT mice. Data are mean ± SEM (n = 3, *p < 0.05, Student’s t test).

We next examined GPR179 and Pikachurin expression and interaction *in vivo*, in the mouse retina. First, we confirmed the ability of the ectodomain of GPR179 to interact with endogenous Pikachurin in a pull-down experiment with mouse retina lysates (Figure 3A). Western blot analysis revealed robust capture of retina-derived Pikachurin by the beads coated with ectoGPR179-Fc protein but not by the control Fc beads, indicating that the binding was specific. Second, we examined immunohistochemical staining of retina cross-sections and found extensive colocalization of Pikachurin and GPR179 in the outer plexiform layer (OPL), where both proteins showed characteristic punctate staining at photoreceptor synapses (Figure 3B). Third, we tested their interaction *in situ*, using a proximity ligation assay (PLA). Using this approach, we found numerous positive signals generated by the antibodies against Pikachurin and GPR179 intersecting at the complex (Figure 3C). The staining pattern corresponded to characteristic synaptic puncta and was confined to the OPL. The puncta predominantly decorated dendritic tips of PKCα-positive rod ON-BC but were also present in PKCα-negative sites, indicating Pikachurin-GPR179 complex formation at both rod and cone synapses. This signal was nearly completely abolished by pre-incubation of sections with purified ecto-GPR179-Fc, a dominant-negative protein competing for Pikachurin binding, indicating specificity of the complex detection strategy (Figure 3D). A quantification of the PLA particles further confirmed the interaction specificity and demonstrated that the complex formation is confined to the photoreceptor synapses in OPL (Figure 3E).

Notably, we found the expression of GPR179 and Pikachurin to be highly regulated during retina development. Being undetectable until P11, both proteins are massively induced at the onset of photoreceptor synapse development (P11–P14), peaking with maturation (P21) (Figures 3F and 3G). This induction was accompanied by their accumulation in photoreceptor synapses, reaching maximum at P14 (Figures 3H and 3I). Intriguingly, our quantitative analysis reveals that during early stages synaptic accumulation of Pikachurin precedes that of GPR179, despite trailing it in expression induction (Figures 3F–3I), suggesting that Pikachurin may localize to synapses in a manner independent from GPR179. To test this model, we evaluated the localization of Pikachurin in retinas from *Gpr179*^*nob5*^ mice. This mouse model contains a well-characterized loss-of-function mutation in *Gpr179* that abolishes its protein expression, with no effects on localization of key signaling molecules mGluR6 and TRPM1 (Peachey et al., 2012; Ray et al., 2014). Indeed, we observed normal punctate synaptic accumulation of Pikachurin in *Gpr179*^*nob5*^ retinas (Figure 3H, bottom). This suggests that GPR179-Pikachurin complex assembly at synapses occurs sequentially, with Pikachurin occupying the pre-synaptic site before GPR179 is recruited post-synaptically. In summary, these results establish specific association of GPR179 with the ECM protein Pikachurin at the photoreceptor synapses and show a hierarchical nature of their synaptic targeting initiated by Pikachurin, which accumulates at synapses independently from GPR179.

### Knockout of Pikachurin Disrupts Stability and Postsynaptic Targeting of GPR179

**Figure 4.**
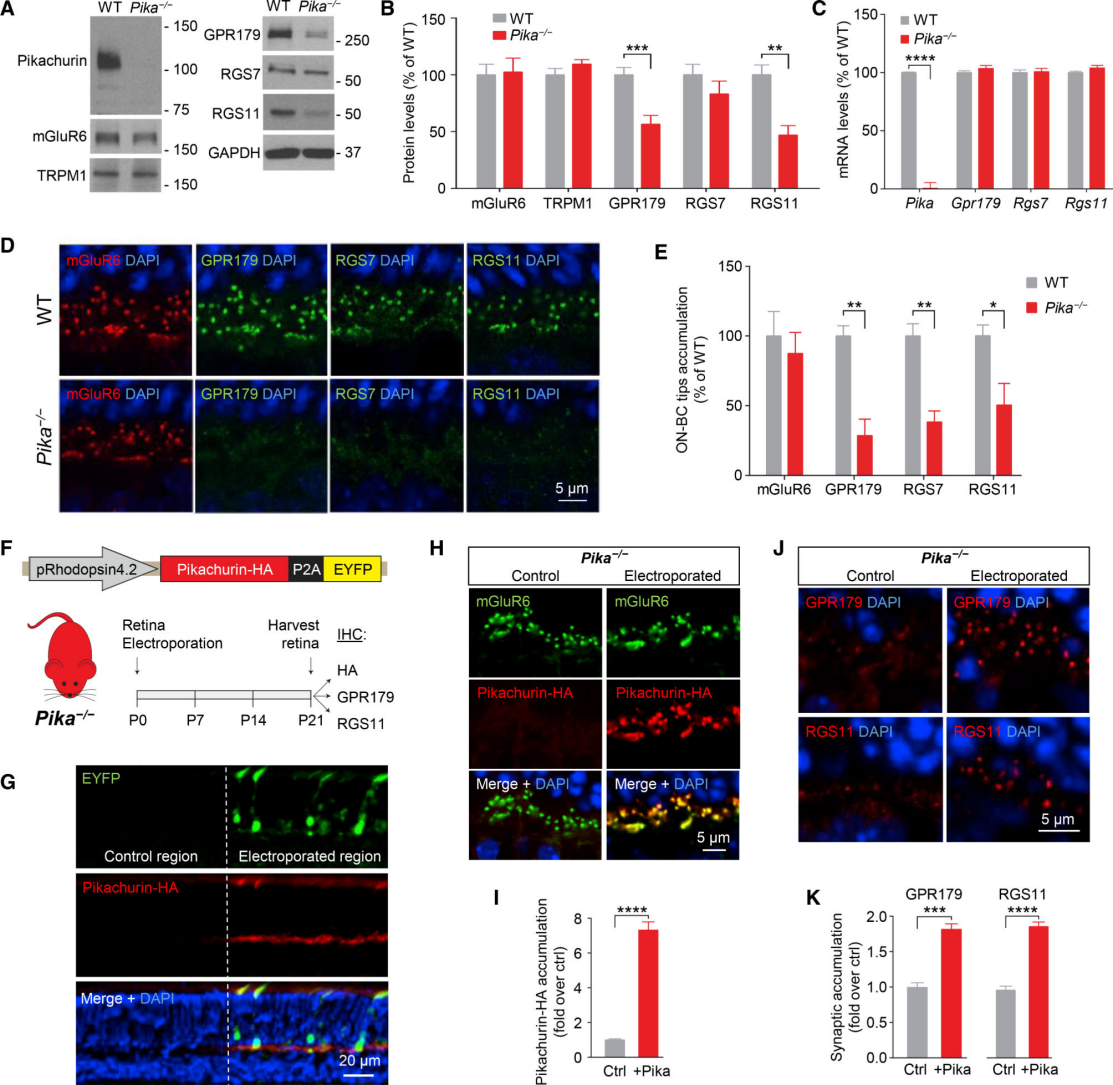
Role of Pikachurin in the Targeting of the Post-synaptic Complex GPR179-RGS Proteins. (A and B) Western blot analysis of the indicated signaling molecules in retina samples from WT and *Pika*^*−/−*^ mice (A) and quantification normalized to GAPDH expression and reported as a percentage of WT (n = 8 WT, n = 5 *Pika*^*−/−*^) (B). (C) qRT-PCR of the indicated genes in WT and *Pika*^*−/−*^ retinas (n = 5 mice/genotype). (D and E) Representative immunohistochemistry of retina sections from WT and *Pika*^*−/−*^ mice using antibodies against mGluR6 (red), GPR179, RGS7, and RGS11 (green) (D) and related quantification (E) (n = 3). (F) *In vivo* retina electroporation experiments. *Pika*^*−/−*^ mice were electroporated at P0 with the outlined photoreceptor-specific construct for Pikachurin overexpression, and the retinas were harvested after 3 weeks and prepared for IHC. (G) Representative confocal image of an individual electroporated retina immunostained with anti-HA (red) and anti-EYFP (green) antibodies. EYFP-negativeregions (left) were used as control for quantification, while EYFP-positive regions (right) represent a successful electroporation. (H and I) Representative confocal images (H) and quantification (I) of synaptic accumulation of overexpressed Pikachurin-HA in control versus electroporated regions. Five to ten different regions of retina from three different mice were used. (J and K) Representative IHC in control and electroporated retina regions in *Pika*^*−/−*^ (J) and quantification of synaptic accumulation (K) of GPR179 (left) and RGS11 (right). DAPI in blue. Five to ten different regions of retina from three different mice were used. Data are mean ± SEM (*p < 0.05, **p < 0.01, ***p < 0.001, and ****p < 0.0001, Student’s t test).

To probe the role of Pikachurin association with GPR179, we analyzed the consequences of eliminating Pikachurin. We found that knockout of Pikachurin in mice (*Pika*^*−/−*^) had no effect on the expression of mGluR6 or TRPM1 (Figures 4A and 4B). Strikingly, we found substantial downregulation in GPR179 expression (Figures 4A and 4B). We further detected significant decrease in the levels of RGS11 and to a lesser extent RGS7 (not statistically significant), which both form a complex with GPR179 (Figures 4A and 4B). This cannot be explained by downregulation of gene expression, as mRNA levels for Gpr179 and Rgs7 and Rgs11 in *Pika*^*−/−*^ retinas were unchanged (Figure 4C), suggesting a role for Pikachurin in the stabilization of the GPR179-RGS protein complex.

**Figure 5.**
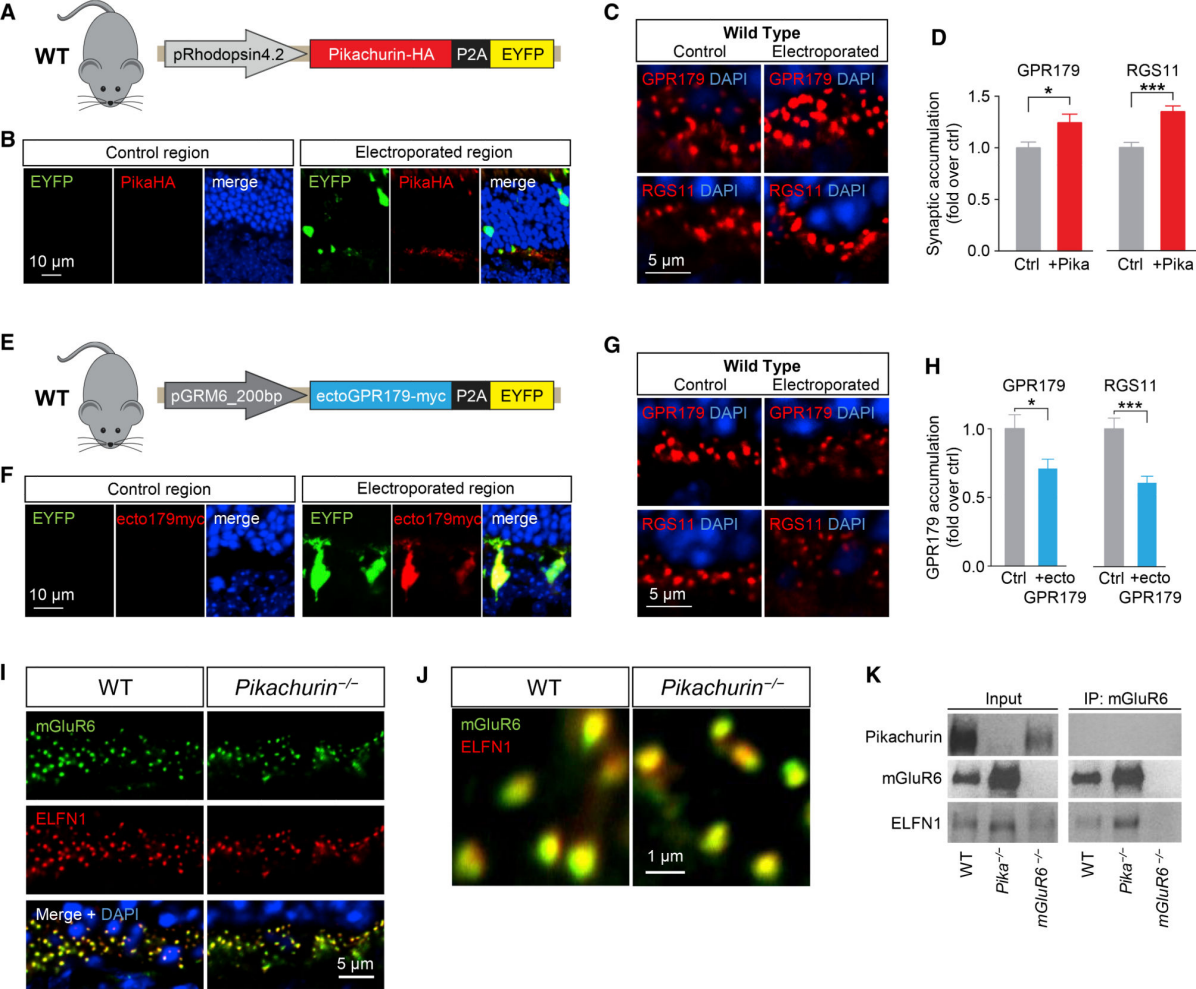
Effects of Cell-Selective Pikachurin Overexpression and Dominant-Negative Blockade of GPR179-Pikachurin Complex Formation in the Retina. (A) Schematics of the construct used for *in vivo* retina electroporation in WT mice. (B) IHC of WT retinas overexpressing Pikachurin-HA in photoreceptors using antibodies against HA (red) or EYFP (green). DAPI in blue. (C and D) IHC of control and electroporated regions in WT mice (C) and quantification of synaptic accumulation (D) of GPR179 (left) and RGS11 (right). DAPI in blue. Five to ten different regions of retina from three different mice were used. (E) Schematics of the dominant-negative construct expressing ectoGPR179myc under control of ON-BC-specific mGluR6 promoter used for in vivo electroporation of WT retinas. (F) IHC using antibodies against EYFP (green) and myc (red). (G and H) Representative IHC in control and electroporated retina regions in WT mice (G) and quantification of synaptic accumulation (H) of GPR179 (left) and RGS11 (right). DAPI in blue. Five to ten different regions of retina from three different mice were used. (I) IHC of mGluR6 (green) and ELFN1 (red) in retina cross-sections of WT and *Pika*^*-/−*^. (J) IHC of mGluR6 and ELFN1 at a higher magnification. (K) CoIP of ELFN1 and mGluR6 in retina samples from WT and *Pika*^*−/−*^ mice. Retinas from *mGluR6*^*−/−*^ mice were used as a control for antibody specificity. Data are mean ± SEM (*p < 0.05 and ***p < 0.001, Student’s t test).

Analysis of synaptic accumulation of proteins by immunostaining of retina sections further revealed deficits in *Pika*^*−/−*^ mice. We found that despite normal localization of mGluR6 (Figures 4D and 4E) originally reported by Omori et al. (2012) and Sato et al. (2008), targeting of GPR179 to the dendritic tips of ON-BC was compromised, as evidenced by substantial reduction in the GPR179-positive synaptic puncta in the OPL (Figures 4D and 4E). Because GPR179 was previously shown to be indispensable for anchoring RGS proteins at the post-synaptic sites (Orlandi et al., 2012), we further examined the localization of RGS7 and RGS11. Consistent with the loss of GPR179 accumulation, we found a significant reduction in the levels of both RGS proteins in the photoreceptor synapses of *Pika*^*−/−*^ retinas (Figures 4D and 4E). To confirm the role of Pikachurin in GPR179 synaptic targeting, we performed a series of rescue, overexpression, and dominant-negative blockade experiments. First, we restored Pikachurin expression selectively in rod photoreceptors of *Pika*^*−/−*^ mice by *in vivo* electroporation delivering HA-tagged full-length Pikachurin directed by rhodopsin promoter (Figure 4F). In photoreceptors expressing the construct, identified by enhanced yellow fluorescent protein (EYFP) fluorescence, ectopic Pikachurin accumulated at synapses recapitulating localization pattern of the native Pikachurin (Figures 4G–4I). Importantly, re-expression of Pikachurin restored synaptic accumulation of GPR179 and RGS11 solely in the positively electroporated regions as confirmed by quantitative analysis (Figures 4J and 4K), indicating that expression of Pikachurin in rods is sufficient in driving post-synaptic accumulation of GPR179RGS7/11 complex. Next, we studied consequences of overexpressing Pikachurin in wild-type mice, adopting the same *in vivo* electroporation strategy (Figures 5A and 5B). We found that increasing Pikachurin expression resulted in increased synaptic accumulation of GPR179 and RGS11 (Figures 5C and 5D), consistent with the model that Pikachurin is a limiting factor determining synaptic targeting of GPR179-RGS complex. We further tested this model by disrupting GPR179-Pikachurin interaction by a dominant-negative strategy expressing the ectodomain of GPR179 in ON-BC to compete with endogenous GPR179 for Pikachurin binding (Figure 5E). The expression of ecto-GPR179 driven by mGluR6 promoter was indeed confined to the ON-BC in electroporated retinas (Figure 5F). Strikingly, we observed a significant reduction in synaptic accumulation of both GPR179 and RGS11 at the dendritic tips of EYFP-positive ON-BC expressing ecto-GPR179 (Figures 5G and 5H), further supporting a critical role of Pikachurin binding in the localization of GPR179 complex.

We next explored selectivity of Pikachurin effects on the organization of transsynaptic complexes given previously reported structural abnormalities of photoreceptor contacts with ON-BC in *Pika*^*−/−*^ retina (Sato et al., 2008). In these studies, we examined the binding and localization of mGluR6-ELFN1 complexes that bridge the synapse via direct interaction (Cao et al., 2015). In *Pika*^*−/−*^ retinas, both ELFN1 and mGluR6 were concentrated at photoreceptor synapses, where they tightly co-localized in a manner indistinguishable from wild-type retinas. (Figures 5I and 5J). Moreover, mGluR6 and ELFN1 robustly co-immunoprecipitated from *Pika*^*−/−*^ retinas, indicating their preserved interaction (Figure 5K). Thus, we conclude that the effects of Pikachurin on synaptic targeting of GPR179 are specific and unlikely caused by structural perturbations that largely preserve molecular environment and interactions at the synaptic cleft. Taken together, these studies indicate that association with Pikachurin plays an essential role in synaptic targeting of GPR179 and assembly of the post-synaptic GAP complex in ON-BC neurons.

### Localization and Function of Pikachurin-GPR179 Assembly Is Controlled by Pre-synaptic DG Complex in Photoreceptors

**Figure 6.**
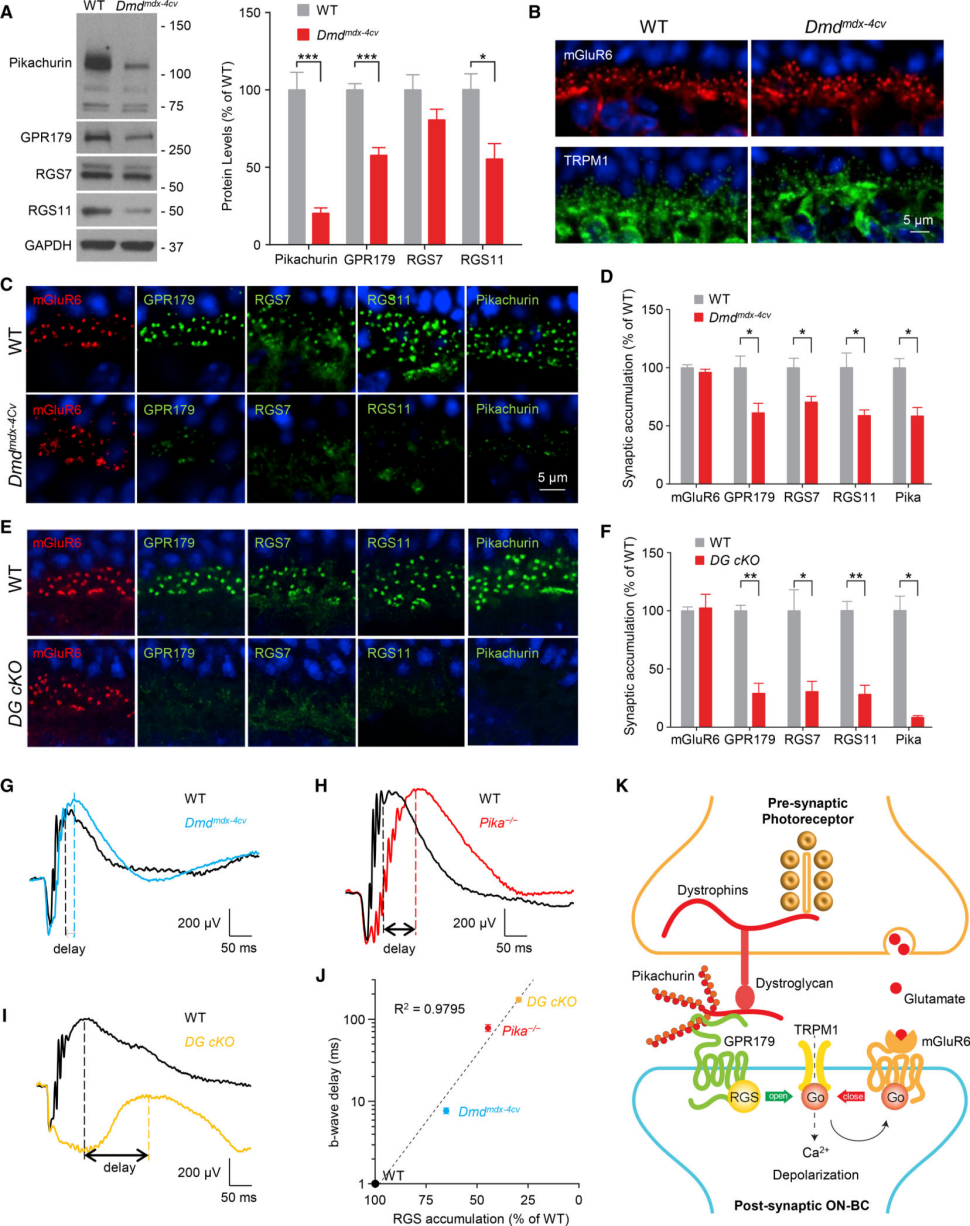
Disruption of the Pre-synaptic DGC Affects Post-synaptic GPR179-RGS Complex Stability and Targeting. (A) Representative western blots and quantification of the indicated proteins in retina samples from WT and Dmdmdx-4Cv mice. Data are mean ± SEM (n = 5 mice/genotype, *p < 0.05 and ***p < 0.001, Student’s t test). (B) Confocal images of retina sections from WT and Dmdmdx-4Cv mice stained with mGluR6 (red) and TRPM1 (green). (C) Representative confocal images of WT and Dmdmdx-4Cv mice stained with antibodies against mGluR6, GPR179, RGS7, RGS11, and Pikachurin. (D) Quantification of mGluR6, GPR179, RGS7, RGS11, and Pikachurin accumulation at the dendritic tips of ON-BCs. Nuclei were stained with DAPI (blue). Data are mean ± SEM (n = 3 mice/genotype, *p < 0.05, Student’s t test). (E) Representative IHC of retina sections from WT and DG cKO mice using antibodies against mGluR6 (red) and GPR179 or RGS7 or RGS11 (green). (F) Quantification of synaptic accumulation of the indicated proteins in WT and DG cKO mice. Data are mean ± SEM (n = 3 mice/genotype, *p < 0.05 and **p < 0.01, Student’s t test). (G–I) Representative traces of ERG responses of dark-adapted WT (black) and (G) Dmdmdx-4Cv (blue; n = 6 WT and n = 7 Dmdmdx-4Cv), (H) Pika−/− (red; n = 3 WT and n = 3 Pika−/−), and (I) DG cKO (orange; n = 3 WT and n = 3 DG cKO) mice at 1 cd s/m2. (J) Correlation between level of RGS protein accumulation (average of RGS7 and RGS11 content quantified from experiments presented in Figure 4E and in D and F) at the dendritic tips of ON-BCs and delay in b-wave implicit time (quantified and averaged from experiments presented in G–I). (K) Schematic model of the transsynaptic macromolecular complex of DGC-Pikachurin and GPR179-RGS proteins.

In the synaptic cleft, Pikachurin is associated with the components of the pre-synaptic DGC in photoreceptors that contain extracellular α-DG, transmembrane β-DG, and intracellular cytosolic dystrophins (Sato et al., 2008). Therefore, we next evaluated the role of these pre-synaptic interactions that tether Pikachurin in the cleft in controlling post-synaptic GPR179 complex. We began our studies by examining *Dmd*^*mdx−4Cv*^ and *Dmd*^*mdx−3Cv*^ mouse models with a partial loss of function in dystrophins compromising the expression of the longest isoforms in photoreceptors (Dp427, Dp260, and Dp140) (Im et al., 1996; Wersinger et al., 2011) and rather mild visual phenotypes (Pillers et al., 1999b; Tsai et al., 2016). Analysis of protein expression by western blotting revealed significantly reduced levels of Pikachurin (20.5 ± 3.2%), GPR179 (57.9 ± 4.4%), and RGS11 (55.4 ±10.0%) in *Dmd*^*mdx−4Cv*^, with a smaller effect on RGS7, which did not reach the level of statistical significance (Figure 6A). A lack of decrease in the corresponding mRNA levels suggests that the observed downregulation is likely caused by destabilization of proteins at the synapse (Figure S2A), similar to findings in *Pika*^*−/−*^ retinas (Figure 4C). We next confirmed downregulation of dystrophins and the previously observed effect on pre-synaptic β-DG in *Dmd*^*mdx−4Cv*^ retinas at photoreceptor synapses by immunostaining (Figure S2B). Strikingly, post-synaptic targeting of GPR179 and associated RGS proteins was substantially compromised (Figures 6C and 6D). We observed no changes in synaptic accumulation of mGluR6 or TRPM1 in *Dmd*^*mdx−4Cv*^ (Figure 6B), again suggesting selective nature of synaptic disturbance. We further detected a significant reduction in the synaptic content of Pikachurin (Figures 6C and S2B), suggesting that the localization deficits are driven by the loss of Pikachurin. Importantly, using *in situ* PLA method, we found that GPR179Pikachurin interaction at the photoreceptor synapses was severely disrupted in *Dmd*^*mdx−4Cv*^ retinas (Figures S2C and S2D). Similar reduction in post-synaptic targeting of GPR179 and RGS proteins was also observed in another model with partial loss in dystrophins, *Dmd*^*mdx−3Cv*^ (Figure S2E), further validating our findings.

To further test the role of transsynaptic interactions, we used a more severe disruption of DGC by ablation of DG using photoreceptor-specific conditional knockout (*DG cKO*) (Omori et al., 2012). *DG cKO* features a nearly complete loss of synaptic Pikachurin (Figures 6E and 6F) amid intact TRPM1 and mGluR6 localization (Omori et al., 2012). Consistent with greater effect on Pikachurin in this model, we found dramatic reduction in the synaptic accumulation of GPR179 (29.0 ± 8.7%), RGS7 (30.4 ± 8.6%), and RGS11 (28.7 ± 7.4%) (Figures 6E and 6F). Together, these observations in *Dmd*^*mdx−4Cv*^, *Dmd*^*mdx−3Cv*^, and *DG cKO* with disruptions in various components of the DGC support the idea that the pre-synaptic DGC is involved in transsynaptic recruitment of post-synaptic GPR179 complex via Pikachurin.

To obtain insight into possible functional consequences of disrupting transsynaptic interactions between DGC and GPR179 complexes, we evaluated synaptic transmission between photoreceptors and ON-BC by electroretinography (ERG) directly comparing functional effects in *Dmd*^*mdx−4Cv*^, *Pika*^*−/−*^, and *DG cKO* models and correlating them with quantitative changes in cytochemical synaptic organization. Specifically, we assessed kinetics of b-wave generation that reflects ON-BC depolarization in response to bright photopic flashes that suppress transmitter release by both rod and cone photoreceptors. The GPR179RGS complex plays an essential role in speeding up the inactivation of Gαo, thus accelerating the depolarizing light response in ON-BCs. Consequently, a reduction in RGS levels at the dendritic tips is well known to result in a progressive slowing of the b-wave onset (Sarria et al., 2015; Shim et al., 2012; Zhang et al., 2010). This suggests that slowing in b-wave kinetics seen upon DGC disruption (Omori et al., 2012; Pillers et al., 1999b; Sato et al., 2008) may be driven by RGS insufficiency. If this were the case, one would expect to observe a correlation between reduction in synaptic RGS content and b-wave deceleration. To test this hypothesis we quantified RGS levels in all three DGC mutants and measured the onset kinetics of b waves side by side using the same ERG paradigm. Indeed, we found that the difference in the implicit times for the b-wave onset tightly correlated with the post-synaptic RGS content: the kinetics were fastest in wild-type (WT) retinas with intact RGS levels and slower in *Dmd*^*mdx−4Cv*^ mice with mild RGS reduction; a larger delay was observed in *Pika*^*−/−*^ mice with a greater reduction in RGS accumulation; and the greatest delay was detected in the *DG cKO* model, in which the synaptic RGS levels were lowest (Figures 6G–6I). In fact, a regression analysis revealed a nearly perfect exponential correlation between these parameters (Figure 6J), strongly supporting our hypothesis. Thus, considering the sum of these observations, we suggest that the transsynaptic coordination between DGC and the major GAP complex of ON-BC is mediated by the Pikachurin-GPR179 complex, which is involved in optimizing the temporal aspects of the synaptic transmission.

## DISCUSSION

In this study, we discover a transsynaptic molecular contact that involves association of DGC with the orphan receptor GPR179. We show that this interaction occurs at the specific synapse between photoreceptors and ON-bipolar neurons and is mediated by the ECM protein Pikachurin. We further demonstrate that Pikachurin recruits the post-synaptic GAP complex, composed of GPR179 and RGS7/11 proteins. Finally, we show that this organization contributes to temporal characteristics of the synaptic transmission. On the basis of our observations, we propose a model in which the HSPG protein Pikachurin, through its C-terminal EGF-like, laminin G domain and HS side chains, acts as a transsynaptic bridge connecting the presynaptic DGC with the extracellular domain of GPR179 on the post-synaptic site. In turn, GPR179 recruits cytoplasmic RGS proteins to control timing of mGluR6 inactivation and therefore the speed of the post-synaptic response to light (Figure 6K). This DGC-Pikachurin-GPR179 complex is a second example of the transsynaptic link at photoreceptor synapses in addition to previously described interaction of mGluR6 with ELFN1 (Cao et al., 2015).

With the tremendous cell-type diversity, one of the key challenges in the nervous system is establishing and maintaining uniqueness of their synaptic communication channels that often require input-specific precision for performing appropriate computations. It is thought that such synaptic specification requires establishing selective transsynaptic contacts involving distinct molecular factors, but this process remains poorly understood, in particular with relevance to functional identity and its heterogeneity (de Wit and Ghosh, 2016). Our findings provide an illustration of a molecular mechanism for specifying unique properties of individual synaptic contacts between well-defined neurons in the retina. Prior studies have demonstrated that molecular interactions between pre-synaptic release apparatus involving Ca_V_1.4 channel complex in photoreceptors and post-synaptic mGluR6 receptor in ON-BC are essential for physical synaptic wiring between these neurons (Cao et al., 2015; Wang et al., 2017). The transsynaptic DGC-GAP bridge reported in this study provides a second channel for the coordination between photoreceptors and ON-BCs. Although it does not play a role in the establishment of physical synaptic contacts of photoreceptors, the DGC-GAP complex specifies the functional properties of this synapse by modulating GPCR signaling.

The Pikachurin-DG complex localized at photoreceptor terminals has been suggested to be involved in visual deficits observed in muscular dystrophy patients (Omori et al., 2012; Sato et al., 2008). In particular, visual deficits affect patients with Duchenne muscular dystrophy with dysfunction in DGC (Fitzgerald et al., 1994; Pillers et al., 1993). These deficits are partially recapitulated in mouse models of the disease carrying mutations in dystrophin gene and prominently include delays in the b-wave onset and visual sensitivity changes (Pillers et al., 1999a, 1999b; Ricotti et al., 2016). Prior studies suggested that deficits in the photoreceptor to ON-bipolar synaptic transmission may be responsible for the phenotype, but the exact molecular mechanisms remained elusive (Green et al., 2004; Omori et al., 2012; Sato et al., 2008; Tsai et al., 2016). Our observations suggest that these synaptic transmission deficits are explained, at least in part, by the dysregulation of the GAP complex consisting of GPR179 and RGS proteins. The levels of RGS protein accumulation at the dendritic tips of ON-bipolar neurons determine the kinetics and sensitivity of their response to changes in photoreceptor inputs (Sarria et al., 2015). Furthermore, complete elimination of RGS7/11 (Cao et al., 2012; Shim et al., 2012) or GPR179 (Audo et al., 2012; Peachey et al., 2012) abolishes the synaptic communication of photoreceptors with ON-bipolar neurons altogether. Our observations show that the DGC is essential for the recruitment of the GAP complex, thus suggesting that the synaptic deficits in Duchenne patients and mouse models originate from disruption in the modulation of the mGluR6 cascade by RGS proteins. In support of this model, we found a strong correlation between varying extent of reduction in GPR179-RGS accumulation at synapses of several DGC deficient models, including *Pika*^*−/−*^, *Dmd*^*Mdx−3cv*^, *Dmd*^*Mdx−4cv*^, and *DG cKO*, and temporal effects on synaptic transmission. Therefore, we think that reduction in GPR179-RGS synaptic accumulation would be sufficient to decelerate synaptic transmission and cause visual deficits in conditions associated with the DGC dysfunction. Nevertheless, it is possible that visual phenotypes in patients with Duchenne muscular dystrophy and related mouse models are not explained solely by the GAP complex disruption. It should be also noted that disruption of DG (Omori et al., 2012) and Pikachurin (Sato et al., 2008) also causes ultrastructural deficits in the organization of the first visual synapse. Thus, it is likely that DGC complex further shapes synaptic transmission and causes functional alterations in photoreceptor communication with ON-bipolar neurons via its effects on structure. However, given that there is no good correlation between the degree of b-wave slowing and structural changes (e.g., loss of RGS or GPR179 severely compromises depolarizing response generation of ON-BC with intact morphology) amid normal molecular architecture of the synaptic cleft (intact TRPM1 and transsynaptic mGluR6-ELFN1 assembly), we think that such changes may play a secondary role in influencing the kinetics of depolarizing response to light and rather affect other aspects of the synaptic transmission. We would like to further note that the severity of the synaptic transmission deficits depends strongly on which component of the pre- or post-synaptic complex is affected, from a mildly delayed b wave with normal amplitude (*Dmd*^*mdx−4Cv*^) to a missing b wave (*Gpr179*^*nob5*^) (Peachey et al., 2012), and intermediate phenotypes for *Pika*^*−/−*^ and *DG cKO* mice, making it possible that other components of DGC further shape this process (Grady et al., 1997).

One of the central observations in this study is the demonstration that orphan receptors GPR158 and GPR179 are direct binding partners of HSPGs. HSPGs form a heterogeneous family of secreted, membrane-bound, and transmembrane components of the ECM that are ubiquitously expressed and regulate a range of biological processes (Bishop et al., 2007; Sarrazin et al., 2011). Importantly, HSPGs have been well documented to play an essential role in the formation and maintenance of synaptic contacts (de Wit et al., 2013; Kamimura et al., 2013; Siddiqui et al., 2013). Thus, the specific case of GPR179-Pikachurin that we evaluated in depth in this study could reflect a more universal mechanism that would generally involve pairing of GPR158/179 with HSPG family members across synaptic contacts in the nervous system. In agreement with this notion, our accompanying study (Condomitti et al., 2018) presents evidence for the role of another such pair GPC4-GPR158 in specifying input-specific synaptic properties in developing hippocampal CA3 pyramidal neurons. We think that further examples on this theme abound and will be of interest to explore in future studies. It will be further interesting to define whether additional elements are present in the HSPG-GPR158/179 complexes. The modular composition of HSPGs and their particular glycosylation pattern are critical in creating a meshwork of transsynaptic proteinprotein interactions responsible for creating gradients of morphogens and growth factors (Baeg et al., 2001; Sudhalter et al., 1996; Yan and Lin, 2009). The interaction with HSPGs prevents these molecules from degradation and diffusion, concentrating them at specific sites (Rosengart et al., 1988; Saksela et al., 1988). Thus, we think it is possible that HSPGs may in fact be co-receptors for GPR158/179 rather than their ligands and act by creating ternary complexes required for further activation of signaling cascades initiated by these receptors.

By establishing extracellular binding partners for the poorly characterized group of orphan receptors GPR158 and GPR179, our study adds to a growing repertoire of such interactions. Most of the known examples come from the “adhesion GPCR” class, whichinthenervoussystemestablishinteractionwithsurfacemolecules to coordinate synaptic development and function (Langenhan et al., 2016). However, members of the class C receptors that feature large ectodomains have also been shown to bind to extracellular proteins (Cao et al., 2015; Tomioka et al., 2014). Our findingsexpandthe range ofthese interactionsand suggestthatassociation with ECM may be a general feature involved incoordinating GPCR function and/or exerting additional regulatory influence.

## STAR★METHODS

### KEY RESOURCES TABLE

**Table:** 

REAGENT or RESOURCE	SOURCE	IDENTIFIER
Antibodies		
Sheep anti-GPR179	Orlandi et al., 2013	N/A
Rabbit anti-RGS11	Cao et al., 2009	N/A
Sheep anti-TRPM1	Cao et al., 2011	N/A
Sheep anti-mGluR6	Cao et al., 2011	N/A
Guinea Pig anti-mGluR6	Koike et al., 2010	N/A
Rabbit anti-RGS7	Gift from Dr. William Simonds (Rojkova et al., 2003)	N/A
Rabbit anti-Dystrophin H4	Gift from Dr. Dominique Mornet (Royuela et al., 2003)	N/A
Rabbit anti-ELFN1	Cao et al., 2015	N/A
Mouse anti-β-DG	Novocastra	Cat# B-DG-CE
Rabbit anti-Pikachurin	Wako	Cat#011–22631
Mouse anti-GAPDH	Millipore	Cat#AB2302; RRID:AB_11211911
Rabbit anti-c-myc	GenScript	Cat#A00172; RRID:AB_914457
Goat anti-Human-Fc-Alexa488	Life Technologies	Cat#A11013
Mouse anti-Human-Fc	Invitrogen	Cat#MA1–10378
Mouse anti-PKCα	Abcam	Cat#ab11723; RRID:AB_298510
Mouse anti-GPR179	Yomics-Primm Biotech	Cat#Ab887; RRID:AB_10792445
Rat anti-HA	Roche	Cat# 11867423001
Rabbit anti-RGS7	Upstate Biotechnology	Cat#07–237
Sheep anti-RGS11	Cao et al., 2009	N/A
Rabbit anti-RFP	Rockland	Cat#600–401-379
Chicken anti-RFP	Rockland	Cat#600–901-379
Chicken anti-GFP	Genscript	Cat#A01694
Chemicals, Peptides, and Recombinant Proteins		
Lipofectamine LTX	Invitrogen	Cat# 15338100
Protein G Sepharose	GE Healthcare	Cat# 17061801
HiTrap Heparin Sepharose	GE Healthcare	Cat# 17–0407-01
Heparinase III	SIGMA	Cat# H8891
Heparin	BEANTOWN CHEMICAL	Cat# 139975
Critical Commercial Assays		
In-Fusion ®HD Cloning kit	Takara Bio USA	Cat# 638910
pcDNA3.1/V5-His TOPO ®TA Expression Kit	Life Technologies	Cat# K480001
Experimental Models: Cell Lines		
HEK293T	ATCC	Cat# CRL-3216, RRID:CVCL_0063
Experimental Models: Organisms/Strains		
Mouse: Pikachurin KO	Sato et al., 2008	N/A
Mouse: Dmd(mdx-3Cv)	(Chapman et al., 1989; Im et al., 1996	N/A
Mouse: Dmd(mdx-4Cv)	Chapman et al., 1989; Im et al., 1996	N/A
Mouse: DG conditional KO	Omori et al., 2012	N/A
Mouse: mGluR6 KO	Riken Bioresource Center	Cat# RBRC05552, RRID:IMSR_RBRC05552
Recombinant DNA		
Full-length GPR158-myc in pcDNA3.1	Orlandi et al., 2012	N/A
Full-length GPR179-myc in pcDNA3.1	Orlandi et al., 2012	N/A
Fc	Cao et al., 2015	N/A
Ecto-GPR158-Fc	This paper	N/A
Ecto-GPR179-Fc	This paper	N/A
GPC1-HA in pcDNA3.1	This paper	N/A
GPC5-HA in pcDNA3.1	This paper	N/A
SDC4-HA in pcDNA3.1	This paper	N/A
Pikachurin-HA in pcDNA3.1	This paper	N/A
pCAG-PikachurinFL-mCherry	Omori et al., 2012	N/A
pCAG-PikachurinLG1–3-mCherry	Omori et al., 2012	N/A
pCAG-PikachurinLG2–3-mCherry	Omori et al., 2012	N/A
pCAG-PikachurinFN-LG2–3-mCherry	Omori et al., 2012	N/A
pCAG-PikachurinFN-mCherry	Omori et al., 2012	N/A
pCAG-PikachurinFN-LG1–2-mCherry	This paper	N/A
pCAG-PikachurinLG3-mCherry	Omori et al., 2012	N/A
pCAG-PikachurinSP-mCherry	Omori et al., 2012	N/A
pRho-Pikachurin-HA-P2A-EYFP	This paper	N/A
pGRM6_200bp-ectoGPR179-myc-P2A-EYFP	This paper	N/A
Software and Algorithms		
Fiji/ImageJ	National Institutes of Health (NIH)	SCR_003070
Zen2.1 SP1 (Black)	Carl Zeiss	SCR_013672
MetaMorph image analysis software	Molecular Devices	SCR_002368
EM LKC Technologies software	Cao et al., 2015	N/A
Prism6	GraphPad Software	SCR_002798
Other		
TaqMan Gene Expression Assay Probe: Egflam	Applied Biosystems	Cat#Mm01298063_m1
TaqMan Gene Expression Assay Probe: Gpr179	Applied Biosystems	Cat#Mm00615352_m1
TaqMan Gene Expression Assay Probe: Rgs7	Applied Biosystems	Cat#Mm01317058_m1
TaqMan Gene Expression Assay Probe: Rgs11	Applied Biosystems	Cat#Mm01309856_m1
TaqMan Gene Expression Assay Probe: Gapdh	Applied Biosystems	Cat#Mm99999915_g1

## CONTACT FOR REAGENT AND RESOURCE SHARING

Further information and requests for resources and reagents should be directed to and will be fulfilled by the Lead Contact, Kirill Martemyanov (kirill@scripps.edu).

## EXPERIMENTAL MODELS AND SUBJECT DETAILS

All experiments were conducted in accordance with the ARVO statement for the use of animals in vision research and guidelines set forth by NIH and approved by the IACUC committee at the Scripps Research Institute. mGluR6^/^ (129S6.129S(Cg)Grm6 < tm1Nak >) were obtained from Riken Bioresource Center (RBRC05552), *Dmd*^*Mdx−4cv*^ and *Dmd*^*Mdx−3cv*^ mice was purchased from The Jackson Laboratory. The generation of *Pika*^*−/−*^, *Gpr179*^*nob5*^, and photoreceptor-specific DG conditional knockout (DG cKO) mice was previously described (Omori et al., 2012; Peachey et al., 2012; Sato et al., 2008). Mice were maintained in a pathogen free facility under standard housing conditions with continuous access to food and water. Mice used in the study were 1–2 months old, and were maintained on a diurnal 12 hr light/dark cycle. No mice displayed health or immune status abnormalities and were not subject to prior procedures. The genotypes of mice are described where appropriate.

## METHOD DETAILS

### Antibodies and genetic constructs

The following commercial antibodies were used: rat anti-HA (Roche), rabbit anti-myc (GenScript), rabbit anti-Pikachurin (Wako), mouse anti-b-DG (Novocastra), mouse anti-PKCα (Abcam), mouse anti-GAPDH (Millipore) mouse anti-human-Fc (ThemoFisher Scientific), chicken anti-GFP (Genscript), rabbit anti-RFP (Rockland) and chicken anti-RFP (Rockland). Mouse anti-GPR179 (YomicsPrimm Biotech) was used for IHC experiments. Rabbit anti-RGS7 (7RC-1), used for IHC on retina sections was a kind gift from Dr. William Simonds (NIDDK, National Institutes of Health, Bethesda) while rabbit anti-RGS7 (Upstate) were used for western blotting. Generation of sheep anti-GPR179, sheep anti-mGluR6, guinea pig anti-mGluR6, sheep anti-TRPM1, rabbit anti-RGS11, rabbit antiELFN1, and Dystrophin H4 (a kind gift from Dr. Dominique Mornet, Universite´ de Montpellier) was previously described (Cao et al., 2009; Cao et al., 2011; Cao et al., 2015; Cao et al., 2008; Koike et al., 2010; Martemyanov et al., 2005; Orlandi et al., 2013; Royuela et al., 2003).

Cloning of full-length mouse GPR158-myc and human GPR179-myc was described (Orlandi et al., 2012). To obtain Fc tagged ecto-GPR158 and ecto-GPR179 expression constructs, the mouse GPR158 (aa 1–417) and human GPR179 (aa 1–380) were subcloned into a previously described pcDM8-derived plasmid expressing the Fc domain of human IgG1 (Farzan et al., 1999) between XhoI and BamHI restriction sites. The control Fc expression construct was the 3CPro expression vector (a gift from Dr. Davide Comoletti, The Child Health Institute of NJ, New Brunswick, NJ, USA). Genetic constructs encoding the full-length sequence of human Pikachurin (GenBank: BC063822.1; cDNA clone MGC:74567), Glypican-1 (GenBank: BC051279.1; cDNA clone MGC:59855), Glypican-5 (GenBank: BC039730.1; cDNA clone MGC:47702) and Syndecan-4 (GenBank: BC030805.1; cDNA clone MGC:22217) were purchased from GE Dharmacon. Each sequence was sub-cloned into pcDNA3.1 vector for mammalian expression and a HA tag was added at the C terminus with In-Fusion HD Cloning technology (Clontech). Plasmids encoding deletions of mouse Pikachurin fused with mCherry in their C terminus were generated by subcloning into pCAG-mCherry backbone. The constructs used for *in vivo* electroporation was generated using In-Fusion HD cloning kit (Clontech) as follows: the human Pikachurin sequence fused with a HA tag in its C terminus and the P2A-EYFP cassette were amplified by PCR reaction and mixed with a PCR-amplified backbone vector containing the 4.4 kb mouse rhodopsin promoter (Lem et al., 1991) to generate the pRho-PikachurinHA-P2A-EYFP vector. The human GPR179 ectodomain sequence (aa 1–380) fused with a myc tag in its C terminus and the P2AEYFP cassette were amplified by PCR reaction and mixed with a PCR-amplified backbone vector containing the mGluR6 promoter, a kind gift from Connie Cepko (Kim et al., 2008), to generate the pGRM6-ectoGPR179myc-P2A-EYFP vector. All constructs were verified by DNA sequencing.

### Quantitative real-time PCR

Total RNA from retina was extracted using TRIZOL reagent (Invitrogen) according to the manufacturer’s instructions. The RNA in the aqueous phase was further purified using RNeasy spin column (QIAGEN). The concentration of purified RNA was obtained with a NanoDrop spectrophotometer (Thermo Fisher Scientific). Reverse transcription was carried out using qScript cDNA Supermix (Quantabio) for qRT-PCR according to manufacturer’s instructions starting from 800 ng of total RNA. The analysis of RNA expression of the target genes was performed on a 7900HT Fast Real-Time PCR System (Applied Biosystems) with Taqman probes under the following conditions: 95°C for 10 min, followed by 40 cycles of 95°C for 15 s, 60°C for 1 min. 5 biological replicates and 3 technical replicates for each sample were used. 16 ng of each sample were used in each real-time PCR (TaqMan Gene Expression Assay ID probes: *Rgs7*: Mm01317058_m1; *Rgs11*: Mm01309856_m1; *Gpr179*: Mm00615352_m1; *Egflam*: Mm01298063_m1; Applied Biosystems). The expression ratio of the target genes was calculated using the *Gapdh* (ID: Mm99999915_g1) as reference using the 2^-ΔΔCT^ method (Livak and Schmittgen, 2001).

### Cell culture, transfection, western blotting, immunoprecipitation, and pull-down assay

HEK293T/17 cells were cultured at 37C and 5% CO_2_ in DMEM supplemented with 10% FBS, MEM non-essential amino acids, 1 mM sodium pyruvate, and antibiotics (100 units/ml penicillin and 100 μg/ml streptomycin). Cells were transfected using Lipofectamine LTX (Invitrogen), harvested 24 h later, lysed in ice-cold IP buffer (300 mM NaCl, 50 mM Tris-HCl pH 7.4, 1% Triton X-100 and complete protease inhibitor cocktail) by sonication. For immunoprecipitation, lysates were cleared by centrifugation at 14,000 g for 15 min, and the supernatants were incubated with 20 μl of Protein G Beads (GE) and 2 μg of antibodies on a rocker at 4°C for 1 h. After three washes with IP buffer, proteins were eluted with 40 μl of 2X SDS sample buffer. Samples were analyzed by SDS-PAGE followed by western blotting using HRP-conjugated secondary antibodies and an ECL West Pico (Thermo Scientific) detection system. For pull-down assays, Fc or ectoGPR179-Fc from transfected HEK293T/17 media were purified by incubation for 1 hr at 4°C with protein G beads. The beads were then incubated for 1 hr at 4°C with lysates of cells expressing the indicated constructs derived from Pikachurin fused to mCherry. Effective pull-down was verified by immunoblotting using antibodies against mCherry (rabbit anti-RFP; Rockland) or Fc fragments (mouse anti-human-Fc; ThemoFisher Scientific).

### Heparinase III treatments

HEK293 cells were transfected with Pika-FL-mCherry, cultured for 16 hr, washed to remove FBS and cultured for additional 24 hr in Optimem. Conditioned medium was collected, concentrated with Amicon filters (30 kDa cutoff) to a final volume of 300 μl and supplemented with (final concentrations) Tris-HCl pH 7.5 (20 mM), CaCl_2_ (4 mM) and Heparinase III (1U/ml; SIGMA). Heparinase III was not added in untreated controls. Samples were incubated for 2h on a rocker at 37°C. In parallel, HEK293 cells transfected with GPR179-myc or pcDNA3.1 as negative control, were prepared for immunoprecipitation using rabbit anti-myc antibodies and conjugated to protein G beads. The beads were then mixed with heparinase III, treated Pikachurin for 1 hr at 4°C, washed 4 times and eluted for western blot analysis.

### Purification of ecto-Fc proteins and mass spectrometry

4 hr after transfection with Fc or GPR-Fc constructs, the medium of HEK293T/17 cells were changed with Optimem. The conditioned Optimem containing the secreted Fc-fused proteins was collected after 72 hr. Dead cells were eliminated by centrifugation and the medium was incubated with 30 ul of Protein G beads (GE) for 1 hr at 4°C. Beads were then washed 3 times with 1% Triton X-100/PBS and retained proteins were eluted with 40 ul of 2X sample buffer (62 mM Tris, 10% glycerol, 2% SDS, and 5% β-mercaptoethanol), entered and SDS-PAGE by applying 1~50 mV for 15–20 min. Gels were fixed with using 5% acetic acid in 50% methanol, stained by NOVEX colloidal blue (Invitrogen). Stained areas were cut out, digested with trypsin (Promega), and alkylated as described previously (Shevchenko et al., 2006). The resulting peptide mixtures were desalted, resolved by high-pressure liquid chromatography, and analyzed using LTQ-Orbitrap XL mass spectrometer, as described previously (Posokhova et al., 2011).

### Immunocytochemistry and Immunohistochemistry

The staining of Pikachurin-HA has been performed on live cells without permeabilization. HEK293 cells were plated on 12 mm coverslips coated with Poly-D-lysine (SIGMA) and transfected with indicated DNA constructs. Transfected cells were incubated overnight at 4°C with rat anti-HA antibody (Roche) in PBS containing 2% donkey serum. After a brief wash with PBS, cells were fixed for 15 min with 4% paraformaldehyde. After three washes with 0.1% Triton X-100/PBS, cells were incubated with Alexa Fluor 546-anti-rat secondary antibodies for 1 hr, washed, and the coverslips where then mounted before mounting in FluoromountDAPI (SouthernBiotech).Eyecups were dissected from mice, fixed for 15 min with 4% paraformaldehyde (PFA), cryoprotected with 30% sucrose in PBS for 2 h at room temperature (RT) and embedded in OCT. 12 μm frozen sections were obtained using a cryostat, permeabilized with 0.1% Triton X-100/PBS for 5 min, blocked with 0.1% Triton X-100/PBS and 10% donkey serum for 1 hr and incubated with primary antibodies in 0.1% Triton X-100/PBS and 2% donkey serum for 1 hr. After 3 washes, sections were incubated with Alexa Fluor 488, 546 or Cy3-conjugated secondary antibodies or Alexa Fluor 647-conjugated PNA for 1 hr. Sections were then washed and mounted using Fluoromount-DAPI (SouthernBiotech).

### Proximity Ligation Assay

Proximity Ligation Assays were performed as previously described (Orlandi et al., 2013). Briefly, sections were prepared, permeabilized, and blocked as for immunohistochemistry and then incubated with primary antibodies (sheep anti-GPR179 1:50; rabbit anti-Pikachurin 1:200 and mouse anti-PKCα 1:100) for 1 hr at RT, followed by 4 washes. The sections were then incubated with Plus-anti-rabbit and Minus-anti-sheep Probemaker-conjugated PLA probes (SIGMA) together with Alexa Fluor 488-conjugated anti-mouse antibody (Invitrogen) in PLA Probe Diluent and Assay Reagent 20X, for 1 hr at 37°C. Sections were washed 4 times, incubated with Ligation-Ligase mix (Ligation Stock 5X with Ligase in high purity water) for 30 min at 37°C, washed 3 times and incubated with Amplification-Polymerase mix (Amplification Stock 5X and Polymerase in high purity water) for 100 min at 37°C. Sections were then washed and mounted with Fluoromount-DAPI (SouthernBiotech). The quantification of PLA particles was performed using ImageJ software. A constant area (4800 μm^2^) was drawn across either the OPL or INL and all positive PLA particles within the area were automatically counted. The total number of bipolar cells that contribute to the formation of these synapses was counted based on DAPI staining and the resulting value was used to normalize the PLA quantification.

### *In vivo* retina electroporation

*In vivo* electroporation was performed as previously described (Matsuda and Cepko, 2004). Briefly, newborn mouse pups were first anesthetized by chilling on ice. A small incision was made in the eyelid and sclera near the lens with a 30-gauge needle. Then ~0.5 μL of DNA solutions (pRho-PikachurinHA-P2A-EYFP, ~5 μg/μl) containing 0.1% fast green were injected sub-retinally using a Hamilton syringe with 32 gauge blunt-ended needle. After injection, tweezer-style electrodes (7 mm Platinum Tweezertrodes, BTX/Harvard Apparatus) applied with electrode gel (Spectra 360, Parker Laboratories, INC.) were placed to clamp softly the head of the pup. 5 square pulses (50 ms duration, 85V, 950 ms intervals) were applied by using a pulse generator (Electro Square Porator, ECM 830, BTX/Harvard Apparatus). Retinas were harvested 3 weeks after electroporation (postnatal day 21), dissected and checked for EYFP expression using fluorescence microscopy (Leica DMI 6000B).

### Confocal Imaging

Images used in this article were generated at The Light Microscopy Facility, the Max Planck Florida Institute, using a Zeiss LSM 880 confocal microscope (Plan-Apochromat 63×/1.4 Oil DIC M27; C-Apochromat 40×/1.2 W Korr FCS M27 and Plan-Apochromat 20×/0.8 M27), at Osaka University on Zeiss LSM700 confocal microscope (alpha Plan-Apochromat 100×/1.46 Oil DIC M27), or at IGBMC Imaging Center using a Leica SP5 microscope (Plan-Apochromat 63×/1.4 Oil) at room temperature. Image acquisition and processing was accomplished using ZEN 2011, ZEN 2009 or LAS-AF software packages with only minor manipulations of the images setting the fluorescence intensity in non-saturating conditions and maintaining similar parameters for each acquired image.

### Bioinformatics

Alignment of GPR158 and GPR179 mouse sequences and identification of a consensus motif were generated using the software ESPript3.0 (Robert and Gouet, 2014). Gene Ontology (GO) enrichment analysis was performed on the list of 129 proteins specifically co-purified with the ecto-GPR158-Fc protein using the Database for Annotation, Visualization and Integrated Discovery (DAVID) tool (Huang et al., 2009). The HSPG Fold Enrichment was calculated as the ratio of number of identified HSPGs on the total number of HSPGs (12/17, 70.59%) and the background information 129/20581 (0.63%) of genes identified.

Multiple alignment of 197 sequences corresponding to Pikachurin across species were performed by a similarity search using the blastp program against the Refseq_protein database (protein sequences from NCBI Reference Sequence project). Parameters used included at least 90% coverage and 50% homology compared to the Pikachurin mouse sequence (NP_001276425.1). Amino acids that were identical in all 197 sequences were highlighted to recognize the most conserved regions.

### Electroretinography

Electroretinograms were recorded as previously described (Omori et al., 2015; Sarria et al., 2015; Yamazaki et al., 2013) using either UTAS (LKC Technologies) or PuREC (Mayo Corporation) systems. Mice were dark-adapted overnight prior to ERG measurements. ERG traces were analyzed using Sigma Plot and Microsoft Excel using previously described approaches (Sarria et al., 2015).

## QUANTIFICATION AND STATISTICAL ANALYSIS

We used Student’s t test to analyze densitometry data from western blot experiments obtained with ImageJ software. Confocal images from immunohistochemistry experiments were used to quantify protein accumulation at the dendritic tips of ON-BCs using Metamorph or ImageJ software packages. A minimum of 3 biological replicates was used for each statistical analysis. Sample sizes ranging from 3 to 6 for biochemical assays (e.g., western blot and IHC) and electrophysiological assays (e.g., ERG) were estimated based on minimum number sufficient to invoke Central Limit Theorem and expected effect sizes observed in previous studies examining the same endpoints. Data from all subjects and samples examined were included with no exclusions. SEM values are provided for each of the plotted mean. Details on particular quantification procedures and analyses are provided in the corresponding section of the STAR Methods section. The confidence values below p < 0.05 were considered to be statistically significant.

## Supplementary Material

1

2

## Highlights

- Orphan receptors GPR158 and GPR179 are membrane partners for HSPGs
- Pikachurin is the endogenous HSPG interacting with GPR179 in photoreceptor synapses
- Loss of Pikachurin disrupts post-synaptic properties via GPR179
- Pikachurin-GPR179 complex assembly is controlled by pre-synaptic dystroglycan complex

## References

[R1] Ahmad R, Wojciech S, and Jockers R (2015). Hunting for the function of orphan GPCRs - beyond the search for the endogenous ligand. Br. J. Pharmacol 172, 3212–3228. 25231237 10.1111/bph.12942 PMC4500361

[R2] Audo I, Bujakowska K, Orhan E, Poloschek CM, Defoort-Dhellemmes S, Drumare I, Kohl S, Luu TD, Lecompte O, Zrenner E, (2012). Whole-exome sequencing identifies mutations in GPR179 leading to autosomal-recessive complete congenital stationary night blindness. Am. J.Hum. Genet 90, 321–330. 22325361 10.1016/j.ajhg.2011.12.007 PMC3276675

[R3] Baeg GH, Lin X, Khare N, Baumgartner S, and Perrimon N (2001). Heparan sulfate proteoglycans are critical for the organization of the extracellular distribution of Wingless. Development 128, 87–94. 11092814 10.1242/dev.128.1.87

[R4] Bargmann CI, and Marder E (2013). From the connectome to brain function.Nat. Methods 10, 483–490. 23866325 10.1038/nmeth.2451

[R5] Bishop JR, Schuksz M, and Esko JD (2007). Heparan sulphate proteoglycans fine-tune mammalian physiology. Nature 446, 1030–1037. 17460664 10.1038/nature05817

[R6] Bolliger MF, Martinelli DC, and Su€dhof TC (2011). The cell-adhesion G protein-coupled receptor BAI3 is a high-affinity receptor for C1q-like proteins.Proc. Natl. Acad. Sci. USA 108, 2534–2539. 21262840 10.1073/pnas.1019577108 PMC3038708

[R7] Cao Y, Song H, Okawa H, Sampath AP, Sokolov M, and Martemyanov KA (2008). Targeting of RGS7/Gbeta5 to the dendritic tips of ON-bipolar cells is independent of its association with membrane anchor R7BP. J. Neurosci 28, 10443–10449. 18842904 10.1523/JNEUROSCI.3282-08.2008 PMC2587022

[R8] Cao Y, Masuho I, Okawa H, Xie K, Asami J, Kammermeier PJ, Maddox DM, Furukawa T, Inoue T, Sampath AP, and Martemyanov KA (2009). Retina-specific GTPase accelerator RGS11/G beta 5S/R9AP is a constitutive heterotrimer selectively targeted to mGluR6 in ON-bipolar neurons. J. Neurosci 29, 9301–9313. 19625520 10.1523/JNEUROSCI.1367-09.2009 PMC2731308

[R9] Cao Y, Posokhova E, and Martemyanov KA (2011). TRPM1 forms complexes with nyctalopin in vivo and accumulates in postsynaptic compartment of ON-bipolar neurons in mGluR6-dependent manner. J. Neurosci 31, 11521–11526. 21832182 10.1523/JNEUROSCI.1682-11.2011 PMC3164511

[R10] Cao Y, Pahlberg J, Sarria I, Kamasawa N, Sampath AP, and Martemyanov KA (2012). Regulators of G protein signaling RGS7 and RGS11 determine the onset of the light response in ON bipolar neurons. Proc. Natl. Acad. Sci. USA 109, 7905–7910. 22547806 10.1073/pnas.1202332109 PMC3356620

[R11] Cao Y, Sarria I, Fehlhaber KE, Kamasawa N, Orlandi C, James KN, Hazen JL, Gardner MR, Farzan M, Lee A, (2015). Mechanism for selective synaptic wiring of rod photoreceptors into the retinal circuitry and its role in vision. Neuron 87, 1248–1260. 26402607 10.1016/j.neuron.2015.09.002 PMC4583715

[R12] Chapman VM, Miller DR, Armstrong D, and Caskey CT (1989). Recovery of induced mutations for X chromosome-linked muscular dystrophy in mice. Proc. Natl. Acad. Sci. USA 86, 1292–1296. 2919177 10.1073/pnas.86.4.1292 PMC286674

[R13] Chia PH, Li P, and Shen K (2013). Cell biology in neuroscience: cellular and molecular mechanisms underlying presynapse formation. J. Cell Biol 203, 11–22. 24127213 10.1083/jcb.201307020 PMC3798257

[R14] Condomitti D, Wierda KD, Schroeder A, Rubio SE, Vennekens KM, Orlandi C, Martemyanov KA, Gounko NV, Savas JN, and de Wit J (2018). An input-specific orphan receptor GPR158-HSPG interaction organizes hippocampal mossy Fiber-CA3 synapses. Neuron 99, Published online 10 2, 2018 10.1016/j.neuron.2018.08.038. PMC6351853 30290982

[R15] de Wit J, and Ghosh A (2016). Specification of synaptic connectivity by cell surface interactions. Nat. Rev. Neurosci 17, 22–35. 26656254 10.1038/nrn.2015.3

[R16] de Wit J, O’Sullivan ML, Savas JN, Condomitti G, Caccese MC, Vennekens KM, Yates JR 3rd, and Ghosh A (2013). Unbiased discovery of glypican as a receptor for LRRTM4 in regulating excitatory synapse development. Neuron 79, 696–711. 23911103 10.1016/j.neuron.2013.06.049 PMC4003527

[R17] Dityatev A, Schachner M, and Sonderegger P (2010). The dual role of the extracellular matrix in synaptic plasticity and homeostasis. Nat. Rev. Neurosci 11, 735–746. 20944663 10.1038/nrn2898

[R18] Farzan M, Mirzabekov T, Kolchinsky P, Wyatt R, Cayabyab M, Gerard NP, Gerard C, Sodroski J, and Choe H (1999). Tyrosine sulfation of the amino terminus of CCR5 facilitates HIV-1 entry. Cell 96, 667–676. 10089882 10.1016/s0092-8674(00)80577-2

[R19] Fitzgerald KM, Cibis GW, Giambrone SA, and Harris DJ (1994). Retinal signal transmission in Duchenne muscular dystrophy: evidence for dysfunction in the photoreceptor/depolarizing bipolar cell pathway. J. Clin. Invest 93, 2425–2430. 8200977 10.1172/JCI117250 PMC294450

[R20] Geiger T, Wehner A, Schaab C, Cox J, and Mann M (2012). Comparative proteomic analysis of eleven common cell lines reveals ubiquitous but varying expression of most proteins. Mol. Cell. Proteomics 11, M111.014050. 10.1074/mcp.M111.014050 PMC3316730 22278370

[R21] Grady RM, Teng H, Nichol MC, Cunningham JC, Wilkinson RS, and Sanes JR (1997). Skeletal and cardiac myopathies in mice lacking utrophin and dystrophin: a model for Duchenne muscular dystrophy. Cell 90, 729–738. 9288752 10.1016/s0092-8674(00)80533-4

[R22] Green DG, Guo H, and Pillers DA (2004). Normal photoresponses and altered b-wave responses to APB in the mdx(Cv3) mouse isolated retina ERG supports role for dystrophin in synaptic transmission. Vis. Neurosci 21, 739–747. 15683561 10.1017/S0952523804215085 PMC1482463

[R23] Huang W, Sherman BT, and Lempicki RA (2009). Systematic and integrative analysis of large gene lists using DAVID bioinformatics resources. Nat. Protoc 4, 44–57. 19131956 10.1038/nprot.2008.211

[R24] Im WB, Phelps SF, Copen EH, Adams EG, Slightom JL, and Chamberlain JS (1996). Differential expression of dystrophin isoforms in strains of mdx mice with different mutations. Hum. Mol. Genet 5, 1149–1153. 8842734 10.1093/hmg/5.8.1149

[R25] Kakegawa W, Mitakidis N, Miura E, Abe M, Matsuda K, Takeo YH, Kohda K, Motohashi J, Takahashi A, Nagao S, (2015). Anterograde C1ql1 signaling is required in order to determine and maintain a single-winner climbing fiber in the mouse cerebellum. Neuron 85, 316–329. 25611509 10.1016/j.neuron.2014.12.020

[R26] Kamimura K, Ueno K, Nakagawa J, Hamada R, Saitoe M, and Maeda N (2013). Perlecan regulates bidirectional Wnt signaling at the Drosophila neuromuscular junction. J. Cell Biol 200, 219–233. 23319599 10.1083/jcb.201207036 PMC3549968

[R27] Kim DS, Matsuda T, and Cepko CL (2008). A core paired-type and POU homeodomain-containing transcription factor program drives retinal bipolar cell gene expression. J. Neurosci 28, 7748–7764. 18667607 10.1523/JNEUROSCI.0397-08.2008 PMC2714707

[R28] Koike C, Obara T, Uriu Y, Numata T, Sanuki R, Miyata K, Koyasu T, Ueno S, Funabiki K, Tani A, (2010). TRPM1 is a component of the retinal ON bipolar cell transduction channel in the mGluR6 cascade. Proc.Natl. Acad. Sci. USA 107, 332–337. 19966281 10.1073/pnas.0912730107 PMC2806705

[R29] Kroeze WK, Sassano MF, Huang XP, Lansu K, McCorvy JD, Gigue` re PM, Sciaky N, and Roth BL (2015). PRESTO-Tango as an open-source resource for interrogation of the druggable human GPCRome. Nat. Struct. Mol. Biol 22, 362–369. 25895059 10.1038/nsmb.3014 PMC4424118

[R30] Langenhan T, Piao X, and Monk KR (2016). Adhesion G protein-coupled receptors in nervous system development and disease. Nat. Rev. Neurosci 17, 550–561. 27466150 10.1038/nrn.2016.86

[R31] Lanoue V, Usardi A, Sigoillot SM, Talleur M, Iyer K, Mariani J, Isope P, Vodjdani G, Heintz N, and Selimi F (2013). The adhesion-GPCR BAI3, a gene linked to psychiatric disorders, regulates dendrite morphogenesis in neurons. Mol. Psychiatry 18, 943–950. 23628982 10.1038/mp.2013.46 PMC3730300

[R32] Lem J, Applebury ML, Falk JD, Flannery JG, and Simon MI (1991). Tissue-specific and developmental regulation of rod opsin chimeric genes in transgenic mice. Neuron 6, 201–210. 1825171 10.1016/0896-6273(91)90356-5

[R33] Lin YC, Boone M, Meuris L, Lemmens I, Van Roy N, Soete A, Reumers J, Moisse M, Plaisance S, Drmanac R, (2014). Genome dynamics of the human embryonic kidney 293 lineage in response to cell biology manipulations. Nat. Commun 5, 4767. 25182477 10.1038/ncomms5767 PMC4166678

[R34] Livak KJ, and Schmittgen TD (2001). Analysis of relative gene expression data using real-time quantitative PCR and the 2(-Delta Delta C(T)) Method. Methods 25, 402–408. 11846609 10.1006/meth.2001.1262

[R35] Luo R, Jeong SJ, Jin Z, Strokes N, Li S, and Piao X (2011). G proteincoupled receptor 56 and collagen III, a receptor-ligand pair, regulates cortical development and lamination. Proc. Natl. Acad. Sci. USA 108, 12925–12930. 21768377 10.1073/pnas.1104821108 PMC3150909

[R36] Manabe R, Tsutsui K, Yamada T, Kimura M, Nakano I, Shimono C, Sanzen N, Furutani Y, Fukuda T, Oguri Y, (2008). Transcriptomebased systematic identification of extracellular matrix proteins. Proc. Natl. Acad. Sci. USA 105, 12849–12854. 18757743 10.1073/pnas.0803640105 PMC2529034

[R37] Marder E (2012). Neuromodulation of neuronal circuits: back to the future.Neuron 76, 1–11. 23040802 10.1016/j.neuron.2012.09.010 PMC3482119

[R38] Martemyanov KA, and Sampath AP (2017). The transduction cascade in retinal ON-bipolar cells: signal processing and disease. Annu. Rev. Vis. Sci 3, 25–51. 28715957 10.1146/annurev-vision-102016-061338 PMC5778350

[R39] Martemyanov KA, Yoo PJ, Skiba NP, and Arshavsky VY (2005). R7BP, a novel neuronal protein interacting with RGS proteins of the R7 family. J. Biol. Chem 280, 5133–5136. 15632198 10.1074/jbc.C400596200

[R40] Matsuda T, and Cepko CL (2004). Electroporation and RNA interference in the rodent retina in vivo and in vitro. Proc. Natl. Acad. Sci. USA 101, 16–22. 14603031 10.1073/pnas.2235688100 PMC314130

[R41] Maurer P, and Hohenester E (1997). Structural and functional aspects of calcium binding in extracellular matrix proteins. Matrix Biol. 15, 569–580. 9138289 10.1016/s0945-053x(97)90033-0

[R42] Morgans CW, Zhang J, Jeffrey BG, Nelson SM, Burke NS, Duvoisin RM, and Brown RL (2009). TRPM1 is required for the depolarizing light response in retinal ON-bipolar cells. Proc. Natl. Acad. Sci. USA 106, 19174–19178. 19861548 10.1073/pnas.0908711106 PMC2776419

[R43] Nitkin RM, Smith MA, Magill C, Fallon JR, Yao YM, Wallace BG, and McMahan UJ (1987). Identification of agrin, a synaptic organizing protein from Torpedo electric organ. J. Cell Biol 105, 2471–2478. 2826489 10.1083/jcb.105.6.2471 PMC2114709

[R44] O’Sullivan ML, de Wit J, Savas JN, Comoletti D, Otto-Hitt S, Yates JR 3rd, and Ghosh A (2012). FLRT proteins are endogenous latrophilin ligands and regulate excitatory synapse development. Neuron 73, 903–910. 22405201 10.1016/j.neuron.2012.01.018 PMC3326387

[R45] Omori Y, Araki F, Chaya T, Kajimura N, Irie S, Terada K, Muranishi Y, Tsujii T, Ueno S, Koyasu T, (2012). Presynaptic dystroglycanpikachurin complex regulates the proper synaptic connection between retinal photoreceptor and bipolar cells. J. Neurosci 32, 6126–6137. 22553019 10.1523/JNEUROSCI.0322-12.2012 PMC6622127

[R46] Omori Y, Kitamura T, Yoshida S, Kuwahara R, Chaya T, Irie S, and Furukawa T (2015). Mef2d is essential for the maturation and integrity of retinal photoreceptor and bipolar cells. Genes Cells 20, 408–426. 25757744 10.1111/gtc.12233

[R47] Ori A, Wilkinson MC, and Fernig DG (2011). A systems biology approach for the investigation of the heparin/heparan sulfate interactome. J. Biol. Chem 286, 19892–19904. 21454685 10.1074/jbc.M111.228114 PMC3103365

[R48] Orlandi C, Posokhova E, Masuho I, Ray TA, Hasan N, Gregg RG, and Martemyanov KA (2012). GPR158/179 regulate G protein signaling by controlling localization and activity of the RGS7 complexes. J. Cell Biol 197, 711–719. 22689652 10.1083/jcb.201202123 PMC3373406

[R49] Orlandi C, Cao Y, and Martemyanov KA (2013). Orphan receptor GPR179 forms macromolecular complexes with components of metabotropic signaling cascade in retina ON-bipolar neurons. Invest. Ophthalmol. Vis. Sci 54, 7153–7161. 24114537 10.1167/iovs.13-12907 PMC3813323

[R50] Orlandi C, Xie K, Masuho I, Fajardo-Serrano A, Lujan R, and Martemyanov KA (2015). Orphan receptor GPR158 is an allosteric modulator of RGS7 catalytic activity with an essential role in dictating its expression and localization in the brain. J. Biol. Chem 290, 13622–13639. 25792749 10.1074/jbc.M115.645374 PMC4447942

[R51] Patel N, Itakura T, Gonzalez JM Jr., Schwartz SG, and Fini ME (2013). GPR158, an orphan member of G protein-coupled receptor Family C: glucocorticoid-stimulated expression and novel nuclear role. PLoS ONE 8, e57843. 23451275 10.1371/journal.pone.0057843 PMC3581496

[R52] Peachey NS, Ray TA, Florijn R, Rowe LB, Sjoerdsma T, ContrerasAlcantara S, Baba K, Tosini G, Pozdeyev N, Iuvone PM, (2012). GPR179 is required for depolarizing bipolar cell function and is mutated in autosomal-recessive complete congenital stationary night blindness. Am. J. Hum. Genet 90, 331–339. 22325362 10.1016/j.ajhg.2011.12.006 PMC3276656

[R53] Pillers DA, Bulman DE, Weleber RG, Sigesmund DA, Musarella MA, Powell BR, Murphey WH, Westall C, Panton C, Becker LE, (1993). Dystrophin expression in the human retina is required for normal function as defined by electroretinography. Nat. Genet 4, 82–86. 8513332 10.1038/ng0593-82

[R54] Pillers DA, Fitzgerald KM, Duncan NM, Rash SM, White RA, Dwinnell SJ, Powell BR, Schnur RE, Ray PN, Cibis GW, and Weleber RG (1999a). Duchenne/Becker muscular dystrophy: correlation of phenotype by electroretinography with sites of dystrophin mutations. Hum. Genet 105, 2–9. 10480348 10.1007/s004399900111

[R55] Pillers DA, Weleber RG, Green DG, Rash SM, Dally GY, Howard PL, Powers MR, Hood DC, Chapman VM, Ray PN, and Woodward WR (1999b). Effects of dystrophin isoforms on signal transduction through neural retina: genotype-phenotype analysis of duchenne muscular dystrophy mouse mutants. Mol. Genet. Metab 66, 100–110. 10068512 10.1006/mgme.1998.2784

[R56] Posokhova E, Song H, Belcastro M, Higgins L, Bigley LR, Michaud NA, Martemyanov KA, and Sokolov M (2011). Disruption of the chaperonin containing TCP-1 function affects protein networks essential for rod outer segment morphogenesis and survival. Mol. Cell. Proteomics 10, M110.000570. 10.1074/mcp.M110.000570 PMC3013443 20852191

[R57] Ray TA, Heath KM, Hasan N, Noel JM, Samuels IS, Martemyanov KA, Peachey NS, McCall MA, and Gregg RG (2014). GPR179 is required for high sensitivity of the mGluR6 signaling cascade in depolarizing bipolar cells. J. Neurosci 34, 6334–6343. 24790204 10.1523/JNEUROSCI.4044-13.2014 PMC4004817

[R58] Ricotti V, Ja¨ gle H, Theodorou M, Moore AT, Muntoni F, and Thompson DA (2016). Ocular and neurodevelopmental features of Duchenne muscular dystrophy: a signature of dystrophin function in the central nervous system.Eur. J. Hum. Genet 24, 562–568. 26081639 10.1038/ejhg.2015.135 PMC4929863

[R59] Robert X, and Gouet P (2014). Deciphering key features in protein structures with the new ENDscript server. Nucleic Acids Res. 42, W320–W324. 24753421 10.1093/nar/gku316 PMC4086106

[R60] Rojkova AM, Woodard GE, Huang TC, Combs CA, Zhang JH, and Simonds WF (2003). Ggamma subunit-selective G protein beta 5 mutant defines regulators of G protein signaling protein binding requirement for nuclear localization. J. Biol. Chem 278, 12507–12512. 12551930 10.1074/jbc.M207302200

[R61] Rosenbaum DM, Rasmussen SG, and Kobilka BK (2009). The structure and function of G-protein-coupled receptors. Nature 459, 356–363. 19458711 10.1038/nature08144 PMC3967846

[R62] Rosengart TK, Johnson WV, Friesel R, Clark R, and Maciag T (1988). Heparin protects heparin-binding growth factor-I from proteolytic inactivation in vitro. Biochem. Biophys. Res. Commun 152, 432–440. 2451918 10.1016/s0006-291x(88)80732-0

[R63] Royuela M, Chazalette D, Hugon G, Paniagua R, Guerlavais V, Fehrentz JA, Martinez J, Labbe JP, Rivier F, and Mornet D (2003). Formation of multiple complexes between beta-dystroglycan and dystrophin family products. J. Muscle Res. Cell Motil 24, 387–397. 14677641 10.1023/a:1027309822007

[R64] Saksela O, Moscatelli D, Sommer A, and Rifkin DB (1988). Endothelial cell-derived heparan sulfate binds basic fibroblast growth factor and protects it from proteolytic degradation. J. Cell Biol 107, 743–751. 2971068 10.1083/jcb.107.2.743 PMC2115214

[R65] Sanes JR, and Yamagata M (2009). Many paths to synaptic specificity. Annu. Rev. Cell Dev. Biol 25, 161–195. 19575668 10.1146/annurev.cellbio.24.110707.175402

[R66] Sarin S, Zuniga-Sanchez E, Kurmangaliyev YZ, Cousins H, Patel M, Hernandez J, Zhang KX, Samuel MA, Morey M, Sanes JR, (2018). Role for Wnt signaling in retinal neuropil development: analysis via RNA-seq and in vivo somatic CRISPR mutagenesis. Neuron 98, 109–126.e8. 29576390 10.1016/j.neuron.2018.03.004 PMC5930001

[R67] Sarrazin S, Lamanna WC, and Esko JD (2011). Heparan sulfate proteoglycans. Cold Spring Harb. Perspect. Biol 3, 3. 10.1101/cshperspect.a004952 PMC3119907 21690215

[R68] Sarria I, Pahlberg J, Cao Y, Kolesnikov AV, Kefalov VJ, Sampath AP, and Martemyanov KA (2015). Sensitivity and kinetics of signal transmission at the first visual synapse differentially impact visually-guided behavior. eLife 4, e06358. 25879270 10.7554/eLife.06358 PMC4412108

[R69] Sato S, Omori Y, Katoh K, Kondo M, Kanagawa M, Miyata K, Funabiki K, Koyasu T, Kajimura N, Miyoshi T, (2008). Pikachurin, a dystroglycan ligand, is essential for photoreceptor ribbon synapse formation. Nat. Neurosci 11, 923–931. 18641643 10.1038/nn.2160

[R70] Shekhar K, Lapan SW, Whitney IE, Tran NM, Macosko EZ, Kowalczyk M, Adiconis X, Levin JZ, Nemesh J, Goldman M, (2016). Comprehensive classification of retinal bipolar neurons by single-cell transcriptomics. Cell 166, 1308–1323.e30. 27565351 10.1016/j.cell.2016.07.054 PMC5003425

[R71] Shen Y, Heimel JA, Kamermans M, Peachey NS, Gregg RG, and Nawy S (2009). A transient receptor potential-like channel mediates synaptic transmission in rod bipolar cells. J. Neurosci 29, 6088–6093. 19439586 10.1523/JNEUROSCI.0132-09.2009 PMC2752970

[R72] Shevchenko A, Tomas H, Havlis J, Olsen JV, and Mann M (2006). In-gel digestion for mass spectrometric characterization of proteins and proteomes. Nat. Protoc 1, 2856–2860. 17406544 10.1038/nprot.2006.468

[R73] Shim H, Wang CT, Chen YL, Chau VQ, Fu KG, Yang J, McQuiston AR, Fisher RA, and Chen CK (2012). Defective retinal depolarizing bipolar cells in regulators of G protein signaling (RGS) 7 and 11 double null mice. J. Biol. Chem 287, 14873–14879. 22371490 10.1074/jbc.M112.345751 PMC3340290

[R74] Siddiqui TJ, and Craig AM (2011). Synaptic organizing complexes. Curr. Opin. Neurobiol 21, 132–143. 20832286 10.1016/j.conb.2010.08.016 PMC3016466

[R75] Siddiqui TJ, Tari PK, Connor SA, Zhang P, Dobie FA, She K, Kawabe H, Wang YT, Brose N, and Craig AM (2013). An LRRTM4HSPG complex mediates excitatory synapse development on dentate gyrus granule cells. Neuron 79, 680–695. 23911104 10.1016/j.neuron.2013.06.029

[R76] Sudhalter J, Whitehouse L, Rusche JR, Marchionni MA, and Mahanthappa NK (1996). Schwann cell heparan sulfate proteoglycans play a critical role in glial growth factor/neuregulin signaling. Glia 17, 28–38. 8723840 10.1002/(SICI)1098-1136(199605)17:1<28::AID-GLIA3>3.0.CO;2-3

[R77] Thomas P, and Smart TG (2005). HEK293 cell line: a vehicle for the expression of recombinant proteins. J. Pharmacol. Toxicol. Methods 51, 187–200. 15862464 10.1016/j.vascn.2004.08.014

[R78] Tomioka NH, Yasuda H, Miyamoto H, Hatayama M, Morimura N, Matsumoto Y, Suzuki T, Odagawa M, Odaka YS, Iwayama Y, (2014). Elfn1 recruits presynaptic mGluR7 in trans and its loss results in seizures. Nat. Commun 5, 4501. 25047565 10.1038/ncomms5501

[R79] Tsai TI, Barboni MT, Nagy BV, Roux MJ, Rendon A, Ventura DF, and Kremers J (2016). Asymmetrical functional deficits of ON and OFF retinal processing in the mdx3Cv mouse model of duchenne muscular dystrophy. Invest. Ophthalmol. Vis. Sci 57, 5788–5798. 27792813 10.1167/iovs.16-19432

[R80] Vardi N, and Dhingra A (2014). Mechanistic basis for G protein function in ON bipolar cells. In G Protein Signaling Mechanisms in the Retina, Martemyanov K and Sampath AP, eds. (New York: Springer), pp. 81–98.

[R81] Wang Y, Fehlhaber KE, Sarria I, Cao Y, Ingram NT, Guerrero-Given D, Throesch B, Baldwin K, Kamasawa N, Ohtsuka T, (2017). The auxiliary calcium channel subunit a2d4 is required for axonal elaboration, synaptic transmission, and wiring of rod photoreceptors. Neuron 93, 1359–1374.e6. 28262416 10.1016/j.neuron.2017.02.021 PMC5364038

[R82] Wersinger E, Bordais A, Schwab Y, Sene A, Be´ nard R, Alunni V, Sahel JA, Rendon A, and Roux MJ (2011). Reevaluation of dystrophin localization in the mouse retina. Invest. Ophthalmol. Vis. Sci 52, 7901–7908. 21896869 10.1167/iovs.11-7519

[R83] Yamazaki D, Funato Y, Miura J, Sato S, Toyosawa S, Furutani K, Kurachi Y, Omori Y, Furukawa T, Tsuda T, (2013). Basolateral Mg2+ extrusion via CNNM4 mediates transcellular Mg2+ transport across epithelia:a mouse model. PLoS Genet. 9, e1003983. 24339795 10.1371/journal.pgen.1003983 PMC3854942

[R84] Yan D, and Lin X (2009). Shaping morphogen gradients by proteoglycans.Cold Spring Harb. Perspect. Biol 1, a002493. 10.1101/cshperspect.a002493 PMC2773635 20066107

[R85] Zhang J, Jeffrey BG, Morgans CW, Burke NS, Haley TL, Duvoisin RM, and Brown RL (2010). RGS7 and −11 complexes accelerate the ONbipolar cell light response. Invest. Ophthalmol. Vis. Sci 51, 1121–1129. 19797214 10.1167/iovs.09-4163 PMC2868462

