# Supplementary material for: Transsynaptic Binding of Orphan Receptor GPR179 to Dystroglycan-Pikachurin Complex Is Essential for the Synaptic Organization of Photoreceptors
Source: Cell Rep. Author manuscript; Available in PMC 2018 Oct 26. (PMC6203450; doi:10.1016/j.celrep.2018.08.068)
Supplement: 1 [file NIHMS1509373-supplement-1.pdf]

Cell Reports, Volume *25*

Supplemental Information

Transsynaptic Binding of Orphan Receptor GPR179 to Dystroglycan-Pikachurin Complex Is Essential for the Synaptic Organization of Photoreceptors

Cesare Orlandi, Yoshihiro Omori, Yuchen Wang, Yan Cao, Akiko Ueno, Michel J. Roux, Giuseppe Condomitti, Joris de Wit, Motoi Kanagawa, Takahisa Furukawa, and Kirill A. Martemyanov

SUPPLEMENTAL FIGURES

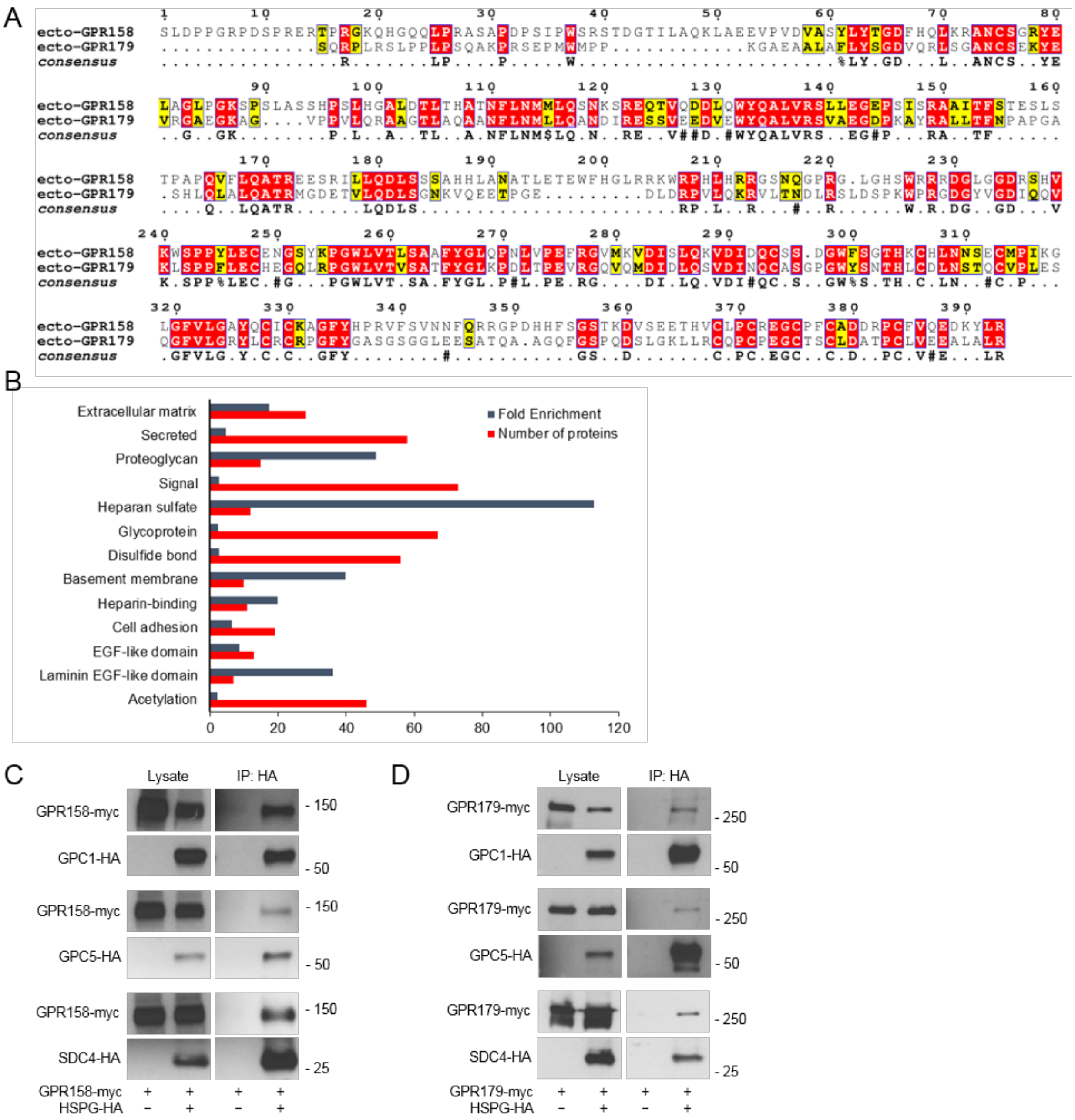

Figure S1 related to Figure 1. Characterization of GPR158/179 interactions with HSPGs.
(**A**) Sequence alignment of mouse GPR158 and GPR179 ectodomains (end of signal peptide to the first transmembrane domain). The consensus motif has been identified with the software ESPript 3.0. Identical amino acids are highlighted in red while amino acids with similar physico-chemical properties in yellow. (**B**) DAVID GO (Database for Annotation, Visualization and Integrated Discovery Gene Ontology) analysis showing the most enriched pathways (False Discovery Rate, FDR<0.01) and the number of proteins in each pathway. (**C**) Immunoprecipitation of several HSPGs using a specific antibody against an HA-tag shows co-immunoprecipitation of myc-tagged GPR158 in co-transfected HEK293 cells. (**D**) Co-IP of HA-tagged HSPGs and myc-tagged GPR179 in co-transfected HEK293 cells.

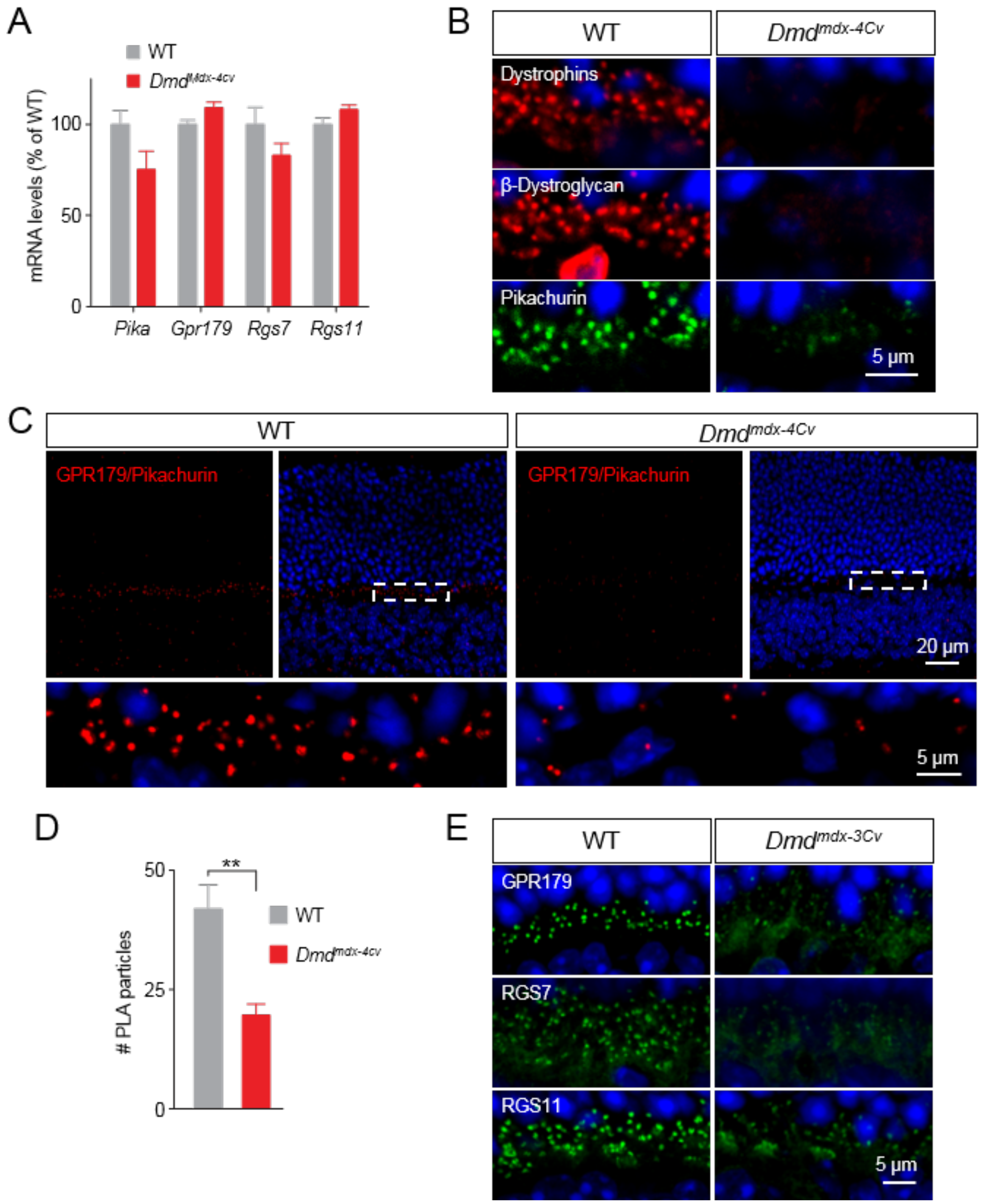

Figure S2 related to Figure 6. **Analysis of protein localization and interactions in DGC loss-of-function models *Dmd*$^{mdx\text{-}4cv}$ and *Dmd*$^{mdx\text{-}3cv}$.** (**A**) Quantitative analysis of the indicated mRNA levels in *Dmd*$^{mdx\text{-}4cv}$ retina samples compared to WT littermates (n = 5 mice/genotype). (**B**) IHC analysis of retina cross-sections of wild type mice compared to *Dmd*$^{mdx\text{-}4cv}$ shows synaptic loss of Dystrophins, β-Dystroglycan and Pikachurin. (**C**) Proximity Ligation Assay of the complex GPR179/Pikachurin in the OPL of WT and *Dmd*$^{mdx\text{-}4cv}$ mice. Red signals represent the complex GPR179/Pikachurin, DAPI staining is in blue. A higher magnification of the OPL region (bottom panel) highlights the reduction in complex formation in *Dmd*$^{mdx\text{-}4cv}$ retina. (**D**) The number of PLA particles/1000 μm^2 of OPL is quantified in the right panel. Data are mean ± SEM (n = 3 mice/genotype. Student's t test; ** P < 0.01). (**E**) Selective reduction in postsynaptic targeting of GPR179/RGS complex in partial Dystrophin loss of function model *Dmd*$^{mdx\text{-}3cv}$. Representative confocal images of retina cross-sections of WT and *Dmd*$^{mdx\text{-}3cv}$ immunolabeled with specific antibodies against GPR179, RGS7 and RGS11. DAPI staining is in blue.
